# Machine learning of neuroimaging for assisted diagnosis of cognitive impairment and dementia: A systematic review

**DOI:** 10.1016/j.dadm.2018.07.004

**Published:** 2018-08-11

**Authors:** Enrico Pellegrini, Lucia Ballerini, Maria del C. Valdes Hernandez, Francesca M. Chappell, Victor González-Castro, Devasuda Anblagan, Samuel Danso, Susana Muñoz-Maniega, Dominic Job, Cyril Pernet, Grant Mair, Tom J. MacGillivray, Emanuele Trucco, Joanna M. Wardlaw

**Affiliations:** a Division of Neuroimaging, Centre for Clinical Brain Sciences and Edinburgh Imaging, University of Edinburgh, Scotland, UK; b Department of Electrical, Systems and Automatics Engineering, Universidad de León, León, Spain; c VAMPIRE project, University of Edinburgh, Scotland, UK; d VAMPIRE project, Computing, School of Science and Engineering, University of Dundee, Dundee, UK; e UK Dementia Institute, University of Edinburgh, Scotland, UK

**Keywords:** Dementia, Cerebrovascular disease, Pathological aging, Small vessel disease, MRI, Machine learning, Classification, Segmentation

## Abstract

**Introduction:**

Advanced machine learning methods might help to identify dementia risk from neuroimaging, but their accuracy to date is unclear.

**Methods:**

We systematically reviewed the literature, 2006 to late 2016, for machine learning studies differentiating healthy aging from dementia of various types, assessing study quality, and comparing accuracy at different disease boundaries.

**Results:**

Of 111 relevant studies, most assessed Alzheimer's disease versus healthy controls, using AD Neuroimaging Initiative data, support vector machines, and only T1-weighted sequences. Accuracy was highest for differentiating Alzheimer's disease from healthy controls and poor for differentiating healthy controls versus mild cognitive impairment versus Alzheimer's disease or mild cognitive impairment converters versus nonconverters. Accuracy increased using combined data types, but not by data source, sample size, or machine learning method.

**Discussion:**

Machine learning does not differentiate clinically relevant disease categories yet. More diverse data sets, combinations of different types of data, and close clinical integration of machine learning would help to advance the field.

## Highlights

• Systematic review of machine learning methods of neuroimaging was performed.
• Machine learning to predict risk of dementia does not seem ready for clinical use.
• Methods have high accuracy to differentiate Alzheimer's disease versus healthy control.
• Performances were poorer when assessing more clinically relevant distinctions.

## 1 Introduction

Aging is associated with increasing health-care costs of which two related neurological disorders, dementia and stroke, account for much of the increase. The total estimated worldwide cost of dementia was US$818 billion in 2015, representing 1.09% of global gross domestic product [1]. In 2015, 46.8 million people worldwide were living with dementia, a figure which is expected to almost double every 20 years, reaching 74.7 million in 2030 and 131.5 million by 2050. Meanwhile, stroke remains the second commonest cause of death and commonest cause of dependency in adults worldwide [2].

Age-related cognitive decline ranges from minor reductions in memory and executive function that do not interfere with daily life to more severe degrees that fall short of dementia but may interfere with some activities of daily living, termed “mild cognitive impairment” (MCI). MCI may progress to dementia or remain static, and cognitive decline is also a risk factor for stroke.

All three MCI, dementia, and stroke are associated with changes seen on brain imaging, particularly brain volume loss (atrophy) and development of focal lesions in the white and gray matter such as white matter hyperintensities (WMH), lacunes, microbleeds, focal cortical or subcortical infarcts, or small hemorrhages. These features are also associated with aging (though are less frequent in healthy aging); may be symptomatic or asymptomatic; and predict increased risk of stroke, dementia, and death [3].

**Fig. 1:**
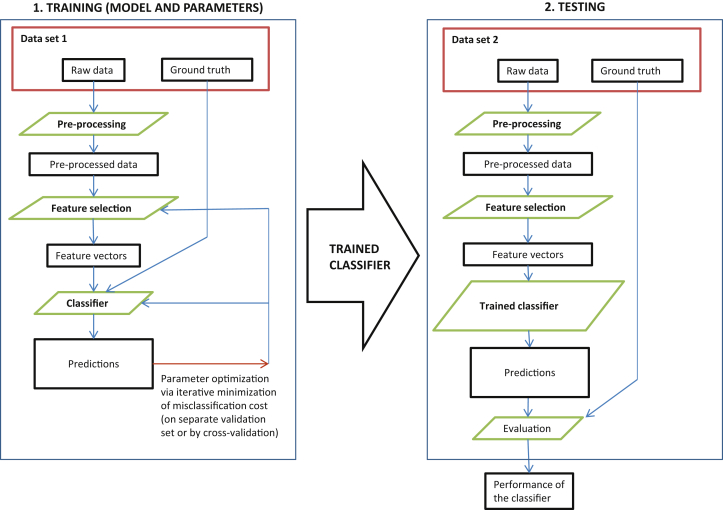
Workflow of traditional (supervised) machine learning studies. For deep learning, feature selection, feature vector, and the classifier to be trained (also preprocessing in some cases) are compressed into a single action (box).

In the last decade, improvements in medical imaging, exponential increase in computational power of affordable computing platforms, and greater availability of neuroimaging data sets, for example, from the Alzheimer's Disease (AD) Neuroimaging Initiative (ADNI), have increased opportunities to develop machine learning approaches to automate detection, classification, and quantification of diseases [4]. Machine learning uses a series of steps to identify, train, and test computer algorithms to identify a feature of interest (Fig. 1). Some of these techniques have been applied to classify brain magnetic resonance imaging or computed tomography scans, comparing patients with dementia and healthy controls, and to distinguish different types or stages of dementia, cerebrovascular disease, and accelerated features of aging. However, the recent rapid increase in publications using different machine learning techniques in different populations, types of images, and disease criteria make it difficult to obtain an objective view of the current accuracy of machine learning.

We undertook this systematic review to critically appraise the accuracy of machine learning to differentiate healthy aging from MCI from dementia and predict the future risk of dementia or cerebrovascular disease. We evaluated the performance metrics of individual machine learning techniques by task, disease of interest, imaging sequence, and features investigated.

## 2 Methods

We performed the review according to Quality Assessment of studies of Diagnostic Accuracy in Systematic reviews (QUADAS), a tool designed for the assessment of published diagnostic studies in systematic reviews, and the Preferred Reporting Items for a Systematic Review and Meta-analysis guidelines. We registered the protocol of this systematic review at the international prospective register of systematic review (PROSPERO, record number: CRD42016037332).

### 2.1 Search Strategy

We searched the literature from January 1, 2006 (when first publications on machine learning in the disorders of interest started appearing in earnest), to September 30, 2016, on six databases: (1) PubMed/Medline; (2) Elsevier; (3) IEEE Xplore Digital Library; (4) Science Direct; (5) Association for Computing Machinery Digital Library; and (6) Web of Science.

We devised three groups of keywords, each relevant to different aspects of the scope of the review:

Brain lesions and relevant pathologies: Dement*, Alzheimer, AD, VCI, VaD, small vessel disease, SVD, microvascular change, cognitive impairment, cognitive decline, MCI, Lewy bod*, LBD, frontotemporal, FTD, lacun*, white matter hyperintens*, white matter lesion*, WMH, leukoaraiosis, periventricular, microbleed*, microhaemorr*, microhemorr*, stroke, cerebrovascular, CVA, perivascular space*, PVS, Virchow–Robin space*, pathological aging, pathological aging, brain, cerebr*, medial temporal, mesial temporal, volume loss, atrophy.
Machine learning: machine learning, supervised learning, unsupervised learning, deep learning, classification, identification, detection, automat* diagnosis, pattern analysis, CAD, computer-aided diagnosis, computer-assisted diagnosis, computational analysis.
Structural imaging: MR, magnetic resonance, structural imag*, CT, CAT, computed tomograph*.
We searched titles, abstracts, and keyword fields of indexed studies published as journal papers or conference proceedings, with all possible strings obtained by joining one term from each of the aforementioned groups with an “AND” operator. One reviewer (E.P.) conducted the searches and eliminated all duplicate references.

### 2.2 Inclusion/exclusion criteria

Two reviewers (E.P. and V.G.C.) separately assessed all nonduplicate papers in a two-stage selection process. First, we evaluated titles and abstracts to exclude studies clearly not relevant to the scope of the review. Second, we assessed full texts of the remaining papers to eliminate studies using the following exclusion criteria:

1. Studies of animals or ex-vivo samples.
2. Reviews, surveys, collections, and comparison papers not presenting a new machine learning method or application.
3. Studies with a validation set comprising a small number of subjects (<100 for disease classification or lesion identification tasks and <25 for pixel- or voxel-level lesion segmentation tasks) or with a manual ground truth provided by only one trained observer.
4. Studies presenting a method in which the main task (e.g., lesion segmentation) was not performed in a fully automated fashion. Studies involving semiautomated preprocessing steps (e.g., brain parcellation refinement) obtained by making use of previously validated software and trained observers were accepted.
5. Studies not about structural magnetic resonance imaging or computed tomography imaging.
6. Studies focused on image preprocessing techniques that did not include any machine learning for disease classification or lesion segmentation/identification (e.g., contrast enhancement, noise reduction techniques, and so forth).
7. Studies of parcellation of healthy brain regions not used for disease classification or detection.
8. Studies that either did not provide or presented their results in such a way that we were not able to calculate performance metrics (e.g., sensitivity and specificity).
9. Multiple publications from the same research group, focusing on the same task and data set. In such cases, only the most recent publication or that with the largest sample size was included in the data analysis.
10. Studies that did not describe their methods in sufficient detail to enable replication.

Discrepancies were resolved by discussion between the two reviewers with a third (M.V.H., L.B., and G.M.) arbitrating as necessary. Notice that none of the studies satisfying the abovementioned criteria reported testing on training data (i.e., either independent training and data sets or proper cross-validation were used); hence, this otherwise necessary exclusion criterion is not included.

### 2.3 Data extraction

From the included papers, we extracted data on the following:

1. disease or lesion investigated,
2. data set used and whether it was publicly available or not,
3. number of subjects or images on which the proposed technique had been validated,
4. type of structural imaging modality and sequences used,
5. imaging features that were investigated,
6. use of any additional imaging data (e.g., functional imaging) or nonimaging features (e.g., cognitive test scores) in the analysis,
7. classifier(s) and the feature selection and representation techniques used, and
8. performance (sensitivity, specificity, and accuracy) of the proposed method.

We extracted data to calculate sensitivity and specificity where they are not already calculated.

If multiple tasks were investigated in a single study, the respective data for each experiment were recorded.

We also extracted (when reported) details of use of single versus multiple scanners, image resolution, population demographics, exclusion criteria for each dataset, image preprocessing steps, time cost, and use of third-party software (details available on request).

We evaluated study quality according to the relevant QUADAS-2 criteria (https://www.ncbi.nlm.nih.gov/pubmed/22007046). We used the seven criteria that were most relevant to the material of the review, four addressing risk of bias and three addressing applicability, because some criteria were not strictly applicable to the field. All acronyms are reported in Supplementary Table 1

### 2.4 Data analysis

We extracted the different performance metrics directly from the papers or calculated them from the data provided. In particular, we aimed to examine the following:

1. Sensitivity, specificity, and accuracy for binary classification tasks.
2. Mean class accuracy for multiclass classification tasks.
3. Dice coefficient for accuracy of lesion segmentation tasks.
4. Precision and recall for lesion identification tasks (calculated using the formula in Supplementary methods).

Where the results of multiple experiments for the same classification task were reported in a single study, we only used the set of metrics associated with the higher value of accuracy in our analysis.

We constructed forest plots to summarize sensitivity, specificity, accuracy, and 95% confidence intervals of various clinically relevant diagnoses including AD versus healthy aging, MCI versus AD or healthy aging, and MCI conversion to AD versus not conversion. To summarize the mass of information effectively, we plotted forest plots of accuracy rather than sensitivity and specificity, which is defined as:

Accuracy=(TP+TN)/(TP+FN+FP+TN)

We performed sensitivity analyses to determine if source data set, machine learning method, type of data used, or study size accounted for the variance between studies. We calculated 95% confidence interval of accuracy using the Wilson [4] score method. We plotted all graphs in R. We considered but rejected performing a formal meta-analysis because the huge overlap in data sets in publications precluded determining the results of patients who contributed to more than one study (even with exclusion of obvious duplicate publications), preventing the modeling of between-study variance. Finally, to minimize confounding by inclusion of studies that only contributed to one comparison, we compared accuracy across multiple diagnostic boundaries using studies that provided data on more than one diagnostic comparison from the same data set.

### 2.5 Role of the Funding Source

The funders had no role in the conduct of this systematic review. The corresponding author confirms that she had full access to all the data in the study and had final responsibility for the decision to submit for publication.

## 3 Results

**Fig. 2:**
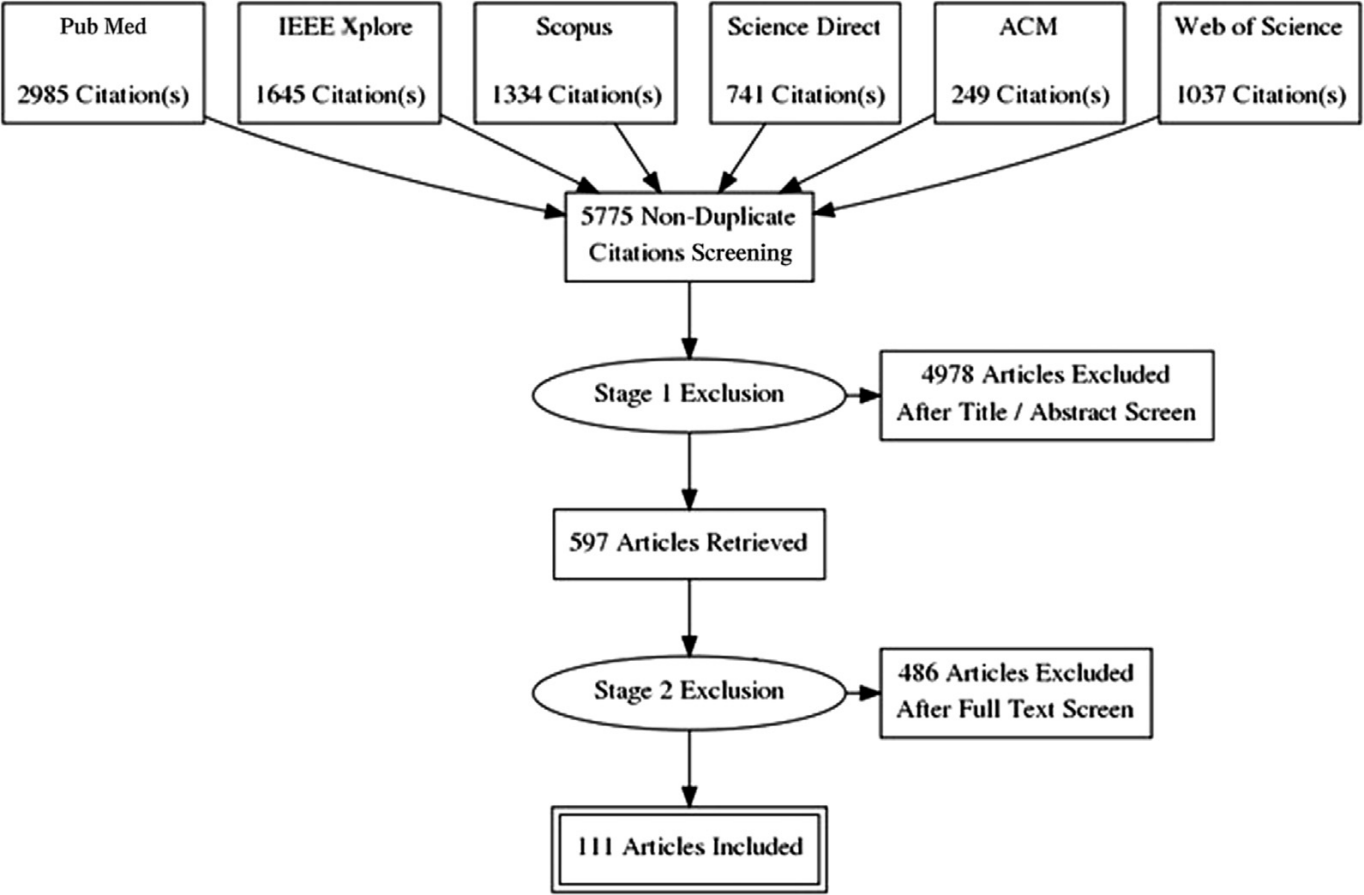
Flowchart of search and exclusion stages of the review.

Our search yielded 5775 nonduplicate studies, of which 4978 (86%) were excluded at title/abstract screening as clearly not relevant to the review. After full-text screening, we found 111 papers relevant for data extraction (Fig. 2). The two criteria accounting for the most exclusions were small sample (item 3) and no performance metrics provided or calculable (item 8; 41% and 19% of exclusions at this stage, respectively; for proportions meeting exclusion criteria see Supplementary Table 2). Note that studies that failed one exclusion criterion were excluded and not evaluated further; although some might have failed on multiple criteria, we only recorded the first reason for exclusion.

Most of the 111 studies that met inclusion criteria achieved low risk of bias scores and low concerns on applicability (Supplementary Table 3; Supplementary Fig. 1). Of the 111, we used 89 studies in further analyses of accuracy where data could be extracted as 2 × 2 tables, and there were sufficient studies to compare.

Most studies tested the diagnosis of AD (68/89, 76%), most versus healthy controls (67/89, 75%), then MCI nonconverters to AD versus converters to AD (37/89, 42%), MCI versus healthy controls (29/89, 33%), and MCI versus AD (8/89, 9%; Table 1 shows individual comparisons; full details are provided in Supplementary Table S3). There were 21 studies that compared multiple diagnostic classes, of which many involved the same author groups. Most studies used structural imaging, although some included other imaging such as diffusion tensor or radioisotope methods (details provided in Supplementary Table 3); however, there were too few such studies and incomplete reporting of accuracy by imaging type to analyze these additional imaging types.

**Table 1:** Number of comparisons in each systematic review analysis group using specified data source, machine learning method, types of imaging and nonimaging data, and by study size

Data sources	HC versus AD	HC versus MCI	MCInc versus MCIc	MCI versus AD	Total
ADNI	54	24	34	7	119
ADNI + Bdx-3C	0	0	1	0	1
AddNeuroMed	1	0	2	0	3
AddNeuroMed + ADNI	2	1	1	0	4
Local	4	3	0	0	7
OASIS	7	2	0	1	10
Total	68∗	30	38	8	144
Machine learning method					
AdaBoost	1	0	1	0	2
Deep Learning	2	2	0	0	4
Gaussian process	0	0	1	0	1
LDA	5	0	5	1	11
Logistic regression	4	0	2	0	6
OPLS	2	1	1	0	4
QDA	0	0	1	0	1
RBF-NN	0	0	1	0	1
Random forest	3	1	3	0	7
SRC	2	1	2	0	5
SVM	39	22	17	7	85
SVM + MKL	3	1	1	0	5
SVM + OPLS	1	0	1	0	2
SVM + random forest	2	1	2	0	5
SVM + SRC	1	1	0	0	2
kNN	3	0	0	0	3
Total	68∗	30	38	8	144
Types of imaging and imaging plus nonimaging data used					
T1w only	46	13	26	6	91
T1w and other imaging data	8	8	2	0	18
T1w and other types of data	8	3	8	1	20
T1w and both other imaging and types of data	6	6	2	1	15
Total	68∗	30	38	8	144
Size of data set (range from 100 to 902 participants)					
150 and under	30	4	9	2	45
151 to 200	4	10	6	0	20
201 to 250	9	4	6	0	19
251 to 300	4	2	3	0	9
Over 300	21	10	14	6	51
Total	68∗	30	38	8	144

Abbreviations: HC, healthy control; AD, Alzheimer's disease; MCI, mild cognitive impairment; nc, nonconverter to AD; T1w, T1-weighted magnetic resonance imaging; c, converter to AD; LDA, linear discriminant analysis; KNN, k-nearest neighbors; OPLS, Orthogonal Projections to Latent Structures; SRC, Sparse Representation Classification.

NOTE. Individual studies contribute to more than one analysis and use more than one data source, machine learning method, combinations of imaging data, and more than one data set (hence more than one sample size in some studies).

∗ In the 68 HC versus AD comparisons, one study is counted twice as it used two different kinds of imaging.

The remaining studies focused on other factors, other types of dementia (five studies; Supplementary Table S4), and studies investigating different types of brain lesions related to dementia, stroke, and pathological aging, either lesion segmentation (seven studies; Supplementary Table S5) or lesion identification (11 studies; Supplementary Table S6). As there were few eligible studies in the latter three categories, it was not possible to undertake any formal comparisons, for example, of DICE coefficients (for WMH, ischemic stroke lesions), precision, or recall values (for microbleeds, lacunes). However, the DICE coefficients for WMH segmentation (four studies, mean n = 81, range, 38–125) ranged from 0.520 to 0.691 and for infarcts (three studies, mean n = 42, range, 30–60) ranged from 0.670 to 0.740 (Supplementary Table S5). The precision/recall values for microbleeds (three studies, mean n = 66, range, 50–81) for precision ranged 0.101 to 0.443 and for recall they were between 0.870 and 0.986; there was one study on lacunes (n = 132) with precision of 0.154 and recall of 0.968 (Supplementary Table S6).

The 76 analyses focused on AD (Supplementary Table S3) amounted to 68 unique references, with huge overlap in authors and data sources between the studies. As using more than one data source, many studies performed more than one comparison of disease classifications with these multiple data sources, hence amounting to 144 different comparisons (Table 1). Of the 144 comparisons, there were 120 uses of ADNI data (ADNI alone 119/144, 83%; ADNI plus other 120/144, 83%), followed by Oasis (10/144, 7%), local sources (7/144, 5%), and AddNeuroMed (alone 3/144, 2%; plus ADNI 4/144, 3%).

The 76 analyses of AD tested nine different machine learning methods. The most frequent, by a large margin, was support vector machine with 46/76 (61%) when alone and 53/76(70%) when combined with another machine learning method, followed by linear discriminant analysis (6/76, 8%), logistic regression (4/76, 5%), and a few testing k-nearest neighbors such as orthogonal projections to latent structures, random forest, or sparse representation classification (Table 1). Most analyses, by a large margin, used only T1 images (91/144, 63%), with modest numbers using T1 plus other sequences, other types of data, or both. Analysis sample sizes ranged from 100 to 902, with similar numbers of analyses including more than 300 subjects (51/144, 35%) or fewer than 150 subjects (45/144, 31%) (Table 1).

**Fig. 3:**
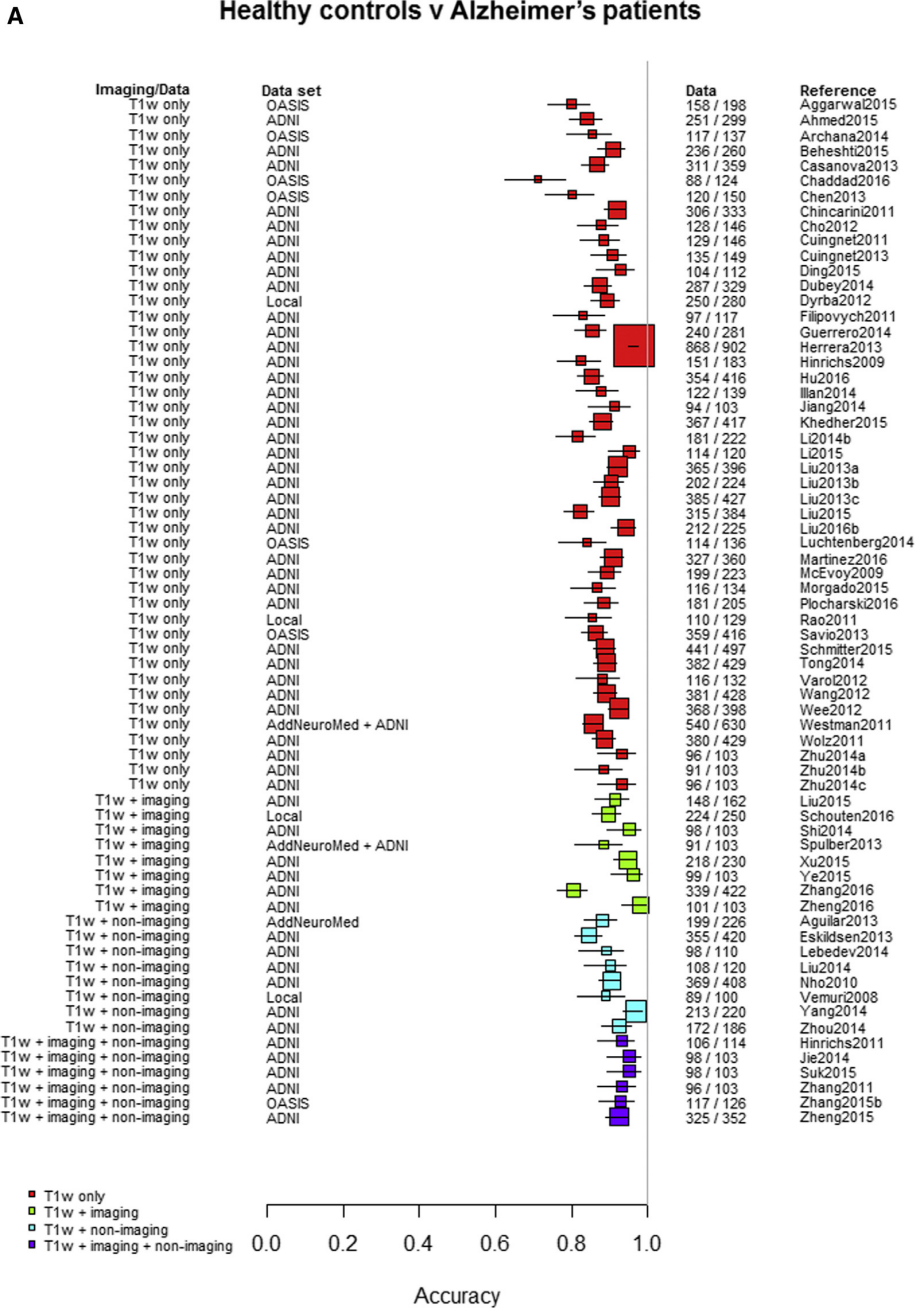

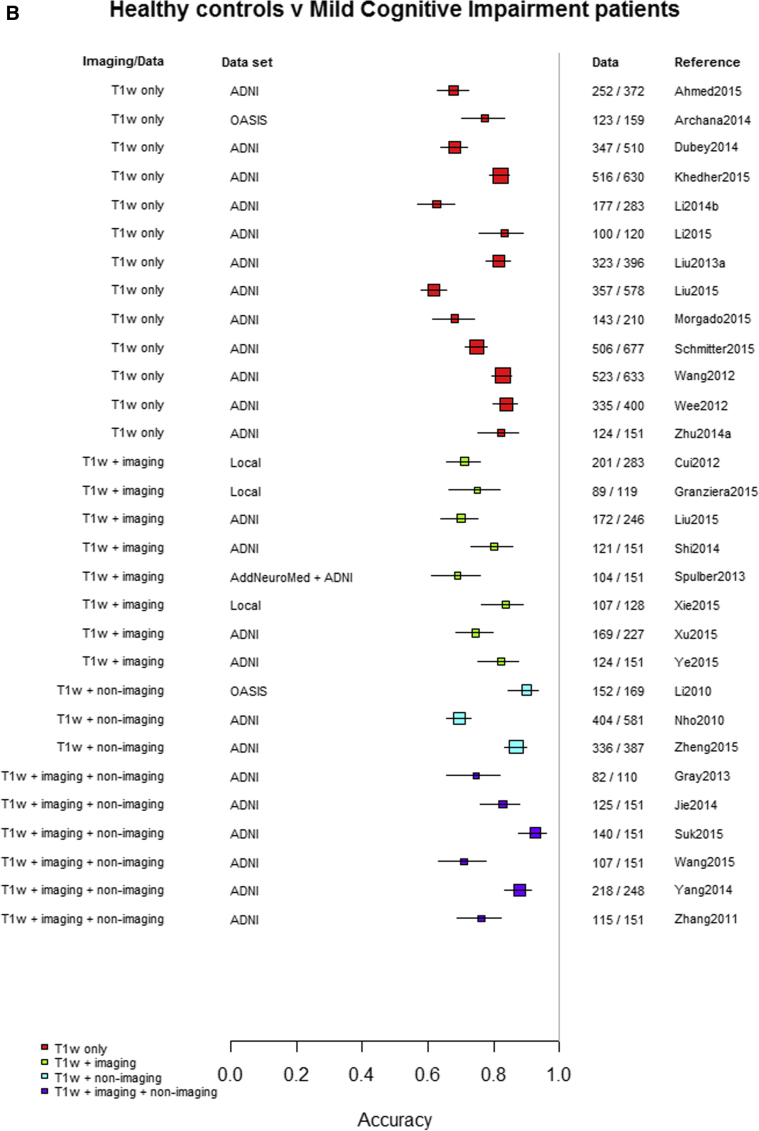

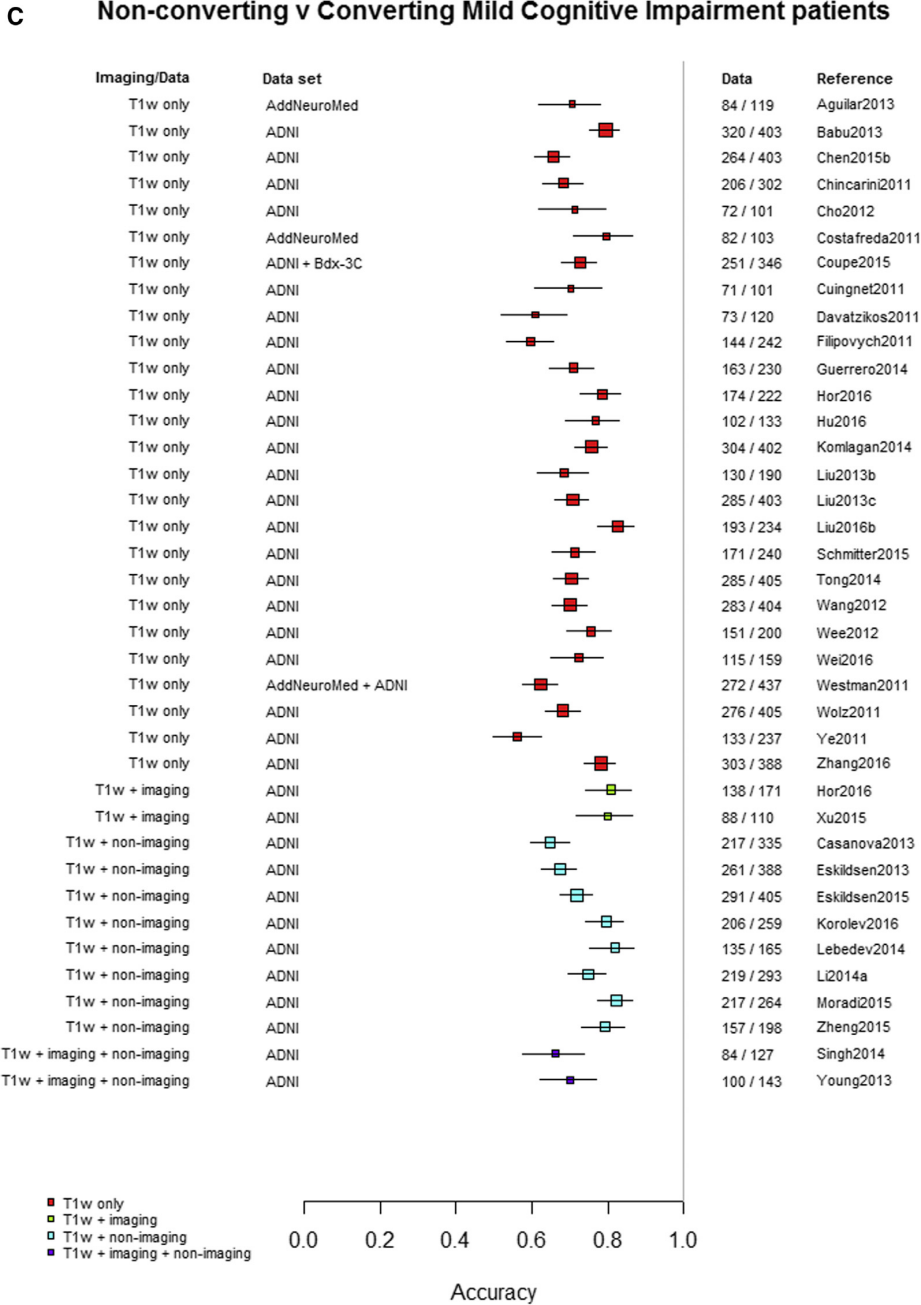

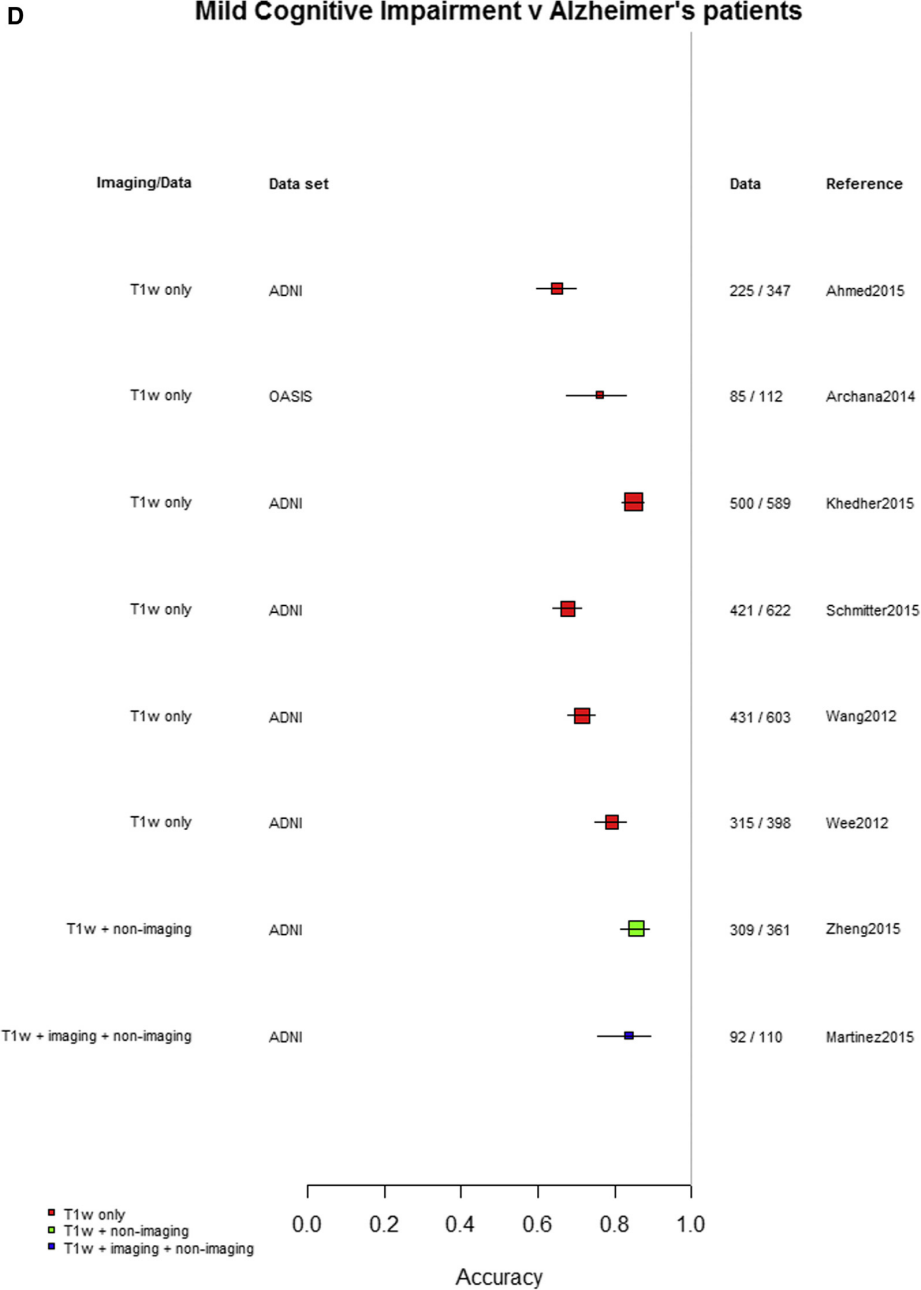
Differentiation of (A) healthy controls from AD, (B) HC from MCI, (C) MCI converters from nonconverters, and (D) MCI from AD, ordered according to the type of data used: T1W only, T1W + other sequences, T1W + nonimaging data, and T1W + other sequences + nonimaging data. Abbreviations: AD, Alzheimer's disease; HC = healthy control; MCI, mild cognitive impairment; T1w, T1-weighted magnetic resonance imaging.

Among the 76 studies focused on AD, the accuracy was higher for differentiating AD from healthy controls (most study accuracies were in the 0.8–1.0 range) than for differentiating MCI from healthy controls (accuracies = 0.6–0.9), nonconverting from converting MCI to AD (accuracies = 0.5–0.85), or MCI from AD (accuracies = 0.6–0.9). Fig. 3A–D indicates the lower accuracy for differentiating healthy controls from MCI, MCI from AD, or MCI nonconverters from converters than healthy controls from AD; Supplementary Figs. 2–4 illustrate these same comparisons ordered by data source, machine learning method, and study size, respectively. There was little evidence of any difference in accuracy by machine learning method, data source used, or study size, with possible higher accuracy for combined T1 plus other sequences and other types of data than for T1 imaging alone.

**Fig. 4:**
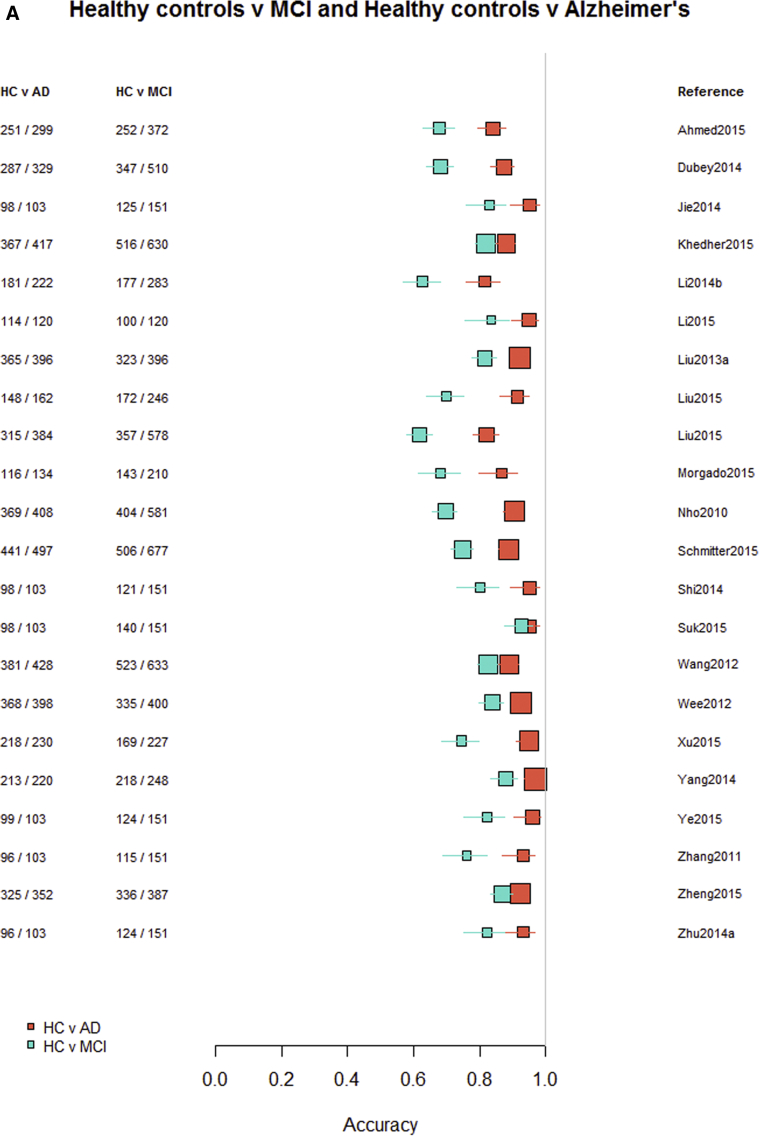

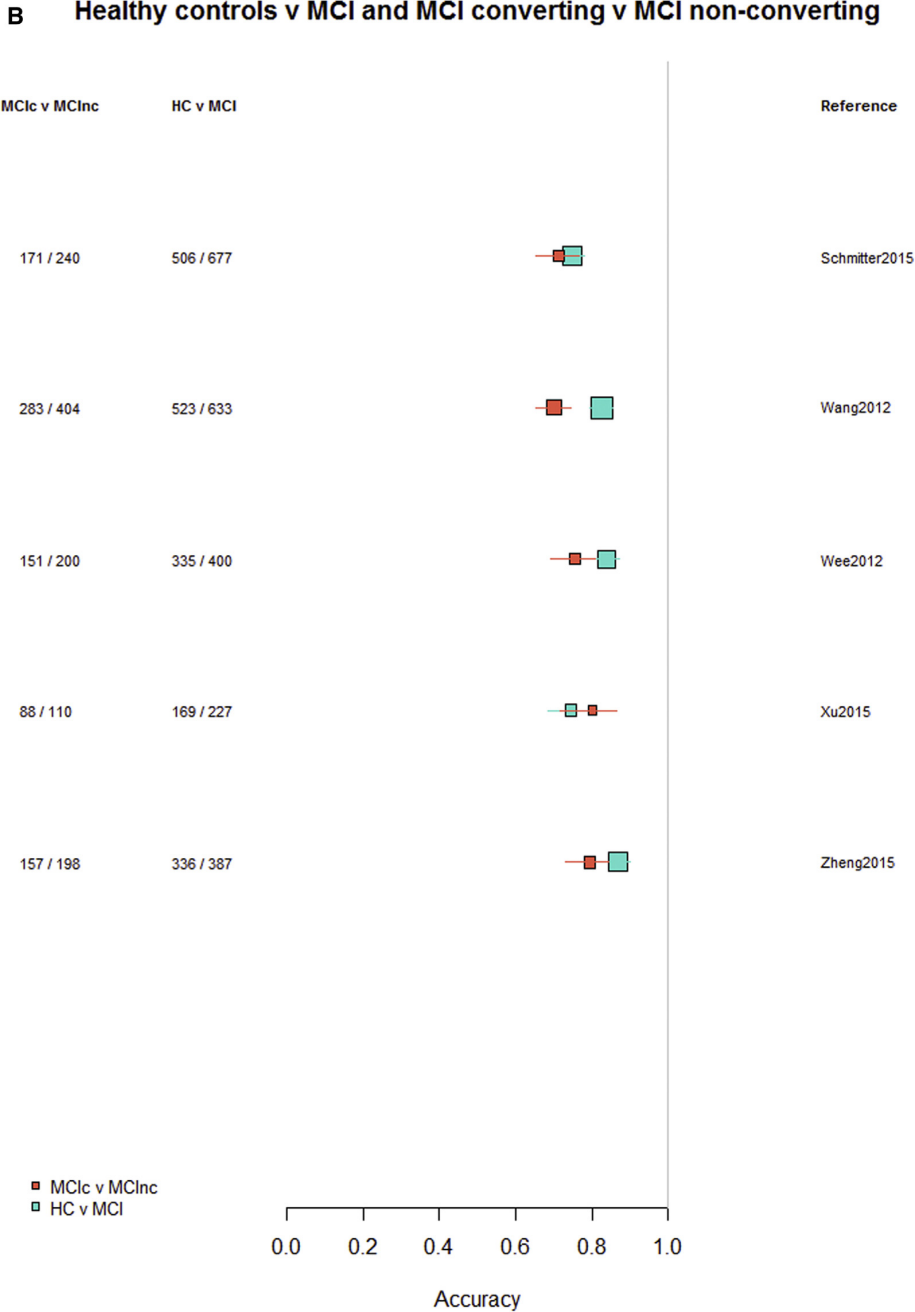

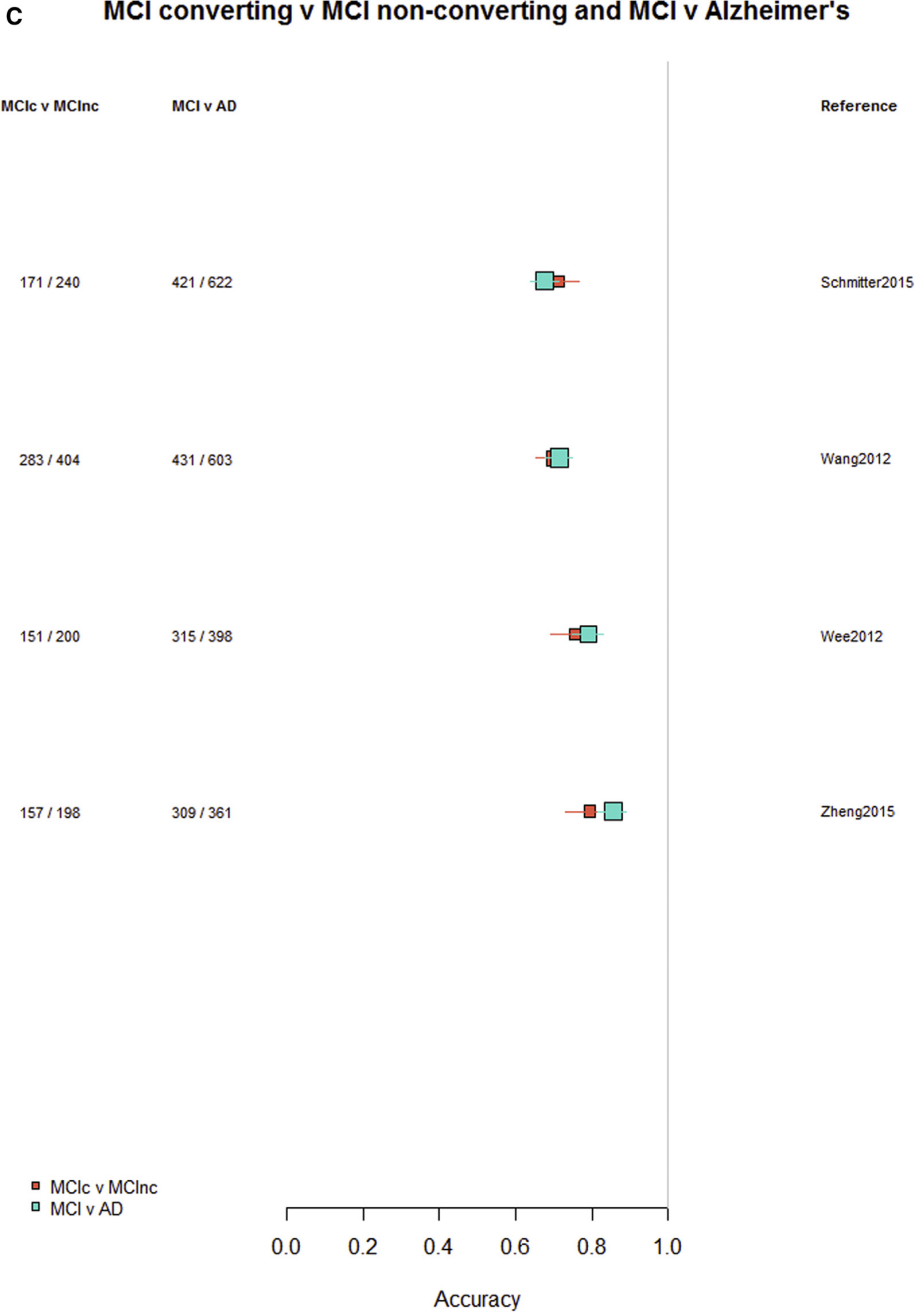

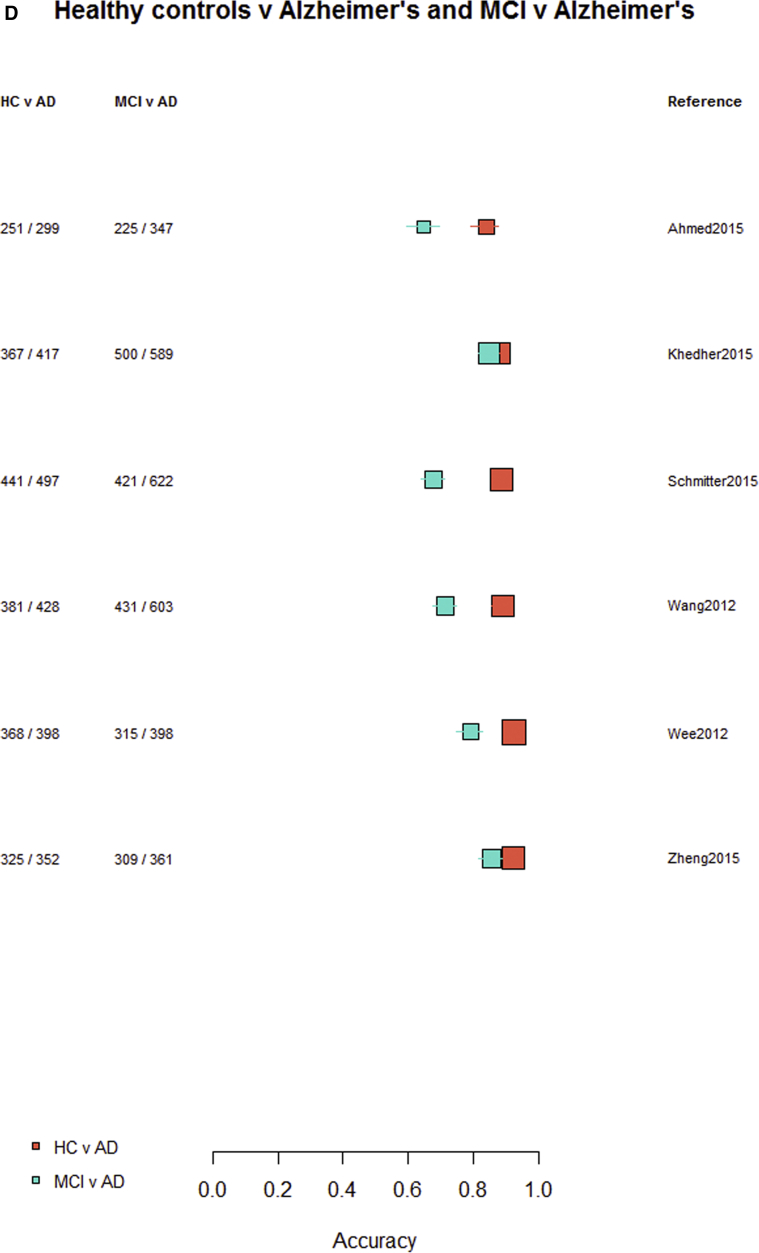
Studies which included more than one diagnostic classification. (A) Healthy controls versus MCI and healthy controls versus AD. (B) Healthy controls versus MCI converting and MCI converting versus MCI nonconverting. (C) MCI converting versus MCI nonconverting and MCI versus AD. (D) Healthy controls versus AD and MCI versus AD. Abbreviations: AD, Alzheimer's disease; MCI, mild cognitive impairment.

Finally, restricting comparisons of accuracy to studies that examined more than one diagnostic classification (Fig. 4A–D) demonstrates the lower accuracy for differentiating between healthy controls and MCI, MCI from AD, or either healthy controls or AD and MCI converting/nonconverting from healthy controls or AD (Fig. 4A–D).

## 4 Discussion

We found acceptable accuracy for all machine learning methods in differentiating healthy controls from AD but fewer data and lower accuracies for differentiating healthy controls from MCI, or MCI from AD, or (more concerning) for risk prediction of MCI nonconverters from converters to AD. From a clinical perspective, the comparison of healthy controls to AD is the least important distinction; such type I diagnostic studies do not produce clinically relevant estimates of sensitivity and specificity but test the initial feasibility of a method. Although the results for machine learning methods in differentiating healthy controls from AD are encouraging, the performance across the other cognitive diagnosis categories indicates that the field has some way to go before these methods should enter routine clinical use [5]. The over-reliance on one data source, populations skewed to the disease of interest with small proportions of controls, one type of imaging, and one machine learning method further limits the clinical relevance and generalizability of the results. This may reflect that, as yet, machine learning is still insufficiently intertwined with the clinical world, in part due to misalignment of targets and methods; although the machine learning community aims primarily for algorithm novelty, inspired largely by computer vision and machine learning, clinicians want reliable, validated methods for early diagnosis, risk prediction, or monitoring interventions, which are better than conventional methods, and change clinical practice.

We aimed to include as many relevant papers as possible, so kept the search broad. We retained conference papers (where sufficient data were reported) to reflect the tendency to publish conference papers that equate to full publications in the fast-moving medical image analysis, computer vision, and machine learning fields. High-quality conferences are at least as selective as many journals; for example, Medical Image Computing and Computer Assisted Intervention, a leading medical image analysis conference, applies a 3-stage selection protocol including rebuttal. About a quarter (29/111, 26%) of the included papers were conference papers. The number of unrefereed preprints becoming available online (e.g., arXiv, biorXiv) is also increasing rapidly, but we did not include these preprint publications because they are not peer-reviewed. However, the use of these sites for dissemination is growing and may need considering in future reviews. The proportion of papers using deep learning has increased since late 2016 (including several published by the authors, many conference papers in MIUA2018 and Medical Image Computing and Computer Assisted Intervention 2017), and therefore, this review may under-represent the most recent developments in machine learning. However, although a brief update of our search to June 2018 found about 100 more papers, most were from the same research groups, published in conference proceedings or ArXiv preprints (therefore would not meet our inclusion criteria), which revealed a substantial expansion in deep learning methods but no obvious shift in accuracy or reporting standards. Many of these recent papers still focused on methods to detect single brain lesion types, such as WMH or atrophy, that are associated with cognitive decline (but not with degrees of cognitive decline itself) or with differentiating AD from healthy controls rather than more subtle diagnoses. It is unlikely that the conclusions of the present analysis, based on a substantial body of work to late 2016, would change by the inclusion of these most recent papers.

Some nonsystematic reviews and surveys on machine learning have been published [6], [7], [8], [9], [10], [11], [12]. Our work included more recent papers, assessed more outcomes, and included sensitivity analyses to assess the impact of key study and population characteristics than prior reviews [13], [14], [15], Applications of deep learning not only in brain but, more in general, in medical imaging have been reviewed in a recently published survey [16]. This work differs from ours in terms of methods (it is not a systematic review) and focus (we did not limit our analysis to deep learning) and scope (we did not include preprints and non–peer-reviewed publications because they lack detail).

We used established systematic review methods, including QUADAS-2 criteria to grade study quality because there are no agreed guidelines for reviews in data science and machine learning. However, we found the QUADAS criteria difficult to apply. We aimed to make reasonable inclusion criteria (publications from 2006 onward, data set larger than 100 for patient/image-level classification, data set larger than 25 for pixel/voxel-level segmentation), based on experience and consultation with experts. We do not believe that the main conclusions would change significantly by including more small studies and also believe that the main messages embedded in the current literature are captured well by the review.

We excluded more than 200 papers (Supplementary Table S2) because the sample size or ground truth annotations were too small. This suggests the need for more public data repositories with annotated, reliable data. Various international initiatives provide public annotated data sets for competitions, e.g. the challenges organized by Medical Image Computing and Computer Assisted Intervention or International Symposium in Biomedical Imaging. Such challenges emphasize the competition aspect (achieving the best values for specific performance parameters), more than maximizing the amount of data made available, the generalizability of the results, or relevance to clinical practice. The latter two should receive more attention if the field is to advance.

We excluded many papers that did not provide accuracy data. This suggests a need to standardize reporting of performance criteria, an issue in the validation of algorithms and software for data and image analysis [17], [18], [19]. Some aspects of the perceived importance of standard criteria and data sets are highlighted by the clear majority of papers using the ADNI data set (www.adni-info.org). Although use of one data set may promote cross-comparisons of results, it is likely to inflate estimates of accuracy and considerably reduces the generalizability of the results to clinical practice. Deep learning techniques are rapidly becoming the methods of choice in medical image analysis and feature in increasing proportions in conferences and journals, for example, many conference papers at MIUA2017. However, the overall message remains the same, i.e., differentiation of AD from healthy controls, but fewer studies and poorer accuracy at differentiating MCI versus healthy control or AD, or MCI converters/nonconverters to AD, with the same problems of sample size, repeated use of the same data and lack of clinical integration. This further increases the need for large data sets as convolutional neural networks have millions of parameters to train. The performance of systems classifying brain images as associated with AD or not seems to improve when using multiple data types [20], [21]. Including nonimaging features, such as CSF biomarkers and cognitive test scores, unsurprisingly also improve performance. Further work is needed to clarify the interplay between data from images and other sources [22].

Most studies started with preprocessed features (“ground truth”) as input to the machine learning method. Many preprocessing techniques used population templates that derive from young populations; these are of limited relevance to the older brain and may bias the resulting outputs [22]. Very few papers on lesion segmentation techniques were included as most failed the inclusion criteria on annotations (ground truth). This reflects that generating sufficient ground truth for a reliable validation of such algorithms is time consuming and highlights a limitation of machine learning methods in relying on ground truth. Use of crowd-sourcing to annotate images may be one solution but would have to achieve high reliability to meet the definition of “ground truth” [23], [24], [25]; their use remains subjudice and depends on the application. We also notice recent work on automatic generation of annotations (auto-annotations) for non∖medical classifiers with large numbers of classes [26] and growing interest of medical image analysts in techniques to minimize the number of annotations required without affecting performance [27].

It proved particularly difficult to locate papers attempting stratification of different types of dementia, and few studies combined imaging with other data types. Possible reasons include that diagnosing dementia is not a clear-cut process, so several covariates should be considered in addition to a binary dementia/no dementia, for example, time of diagnosis, source data for diagnosis (MCI test, brain images, clinical records, prescriptions) while avoiding inappropriate circularity by including variables such as current cognitive test results (several papers may have inflated their estimates of accuracy by including current cognitive test results in their algorithm (Supplementary Table 3) but were too few in number to test the effect in sensitivity analyses. Different dementia components might be present at the same time. Finally, to our best knowledge, no reliably stratified, sufficiently large public neuroimaging data sets exist.

Practically all the included papers were written for a computer science or engineering audience. They focused on technical information (e.g., algorithm, parameter setting, training protocol) omitting essential clinically relevant information (e.g., patient demographics, clinical covariates, data acquisition protocols). To elaborate further, practically all the papers included were written for a computer science or engineering audience. A consequent, but serious, limitation for effective interdisciplinarity is that a clinical audience does not appreciate easily the potential, value, and limits of the methods presented; most technical papers do not address, for instance, issues of patient demographics, disease category, clinical covariates, or data acquisition protocols, which are important for clinicians.* *Specialized journals and conferences require specialist language, but international efforts are needed to make technical papers more understandable to a clinical audience, and vice versa, for example, clinician-oriented summaries addressing the points above, and more.

## 5 Conclusions

Our review indicates that machine learning methods to predict risk of dementia are not yet ready for routine use. Better interdisciplinary collaborations and internationally agreed (by clinicians and computer science/engineers) validation protocols and clinical trials are needed. Development of more machine learning methods in neuroimaging requires much greater interdisciplinary working, varied and clinically relevant annotated data sets, varied imaging types not just T1, and focus on relevant outcomes to ensure that the resulting machine learning methods are robust and reliable before testing in clinical trials.

**Research in Context**

1. Systematic review: The authors searched six databases for machine learning studies published between 2006 and late 2016, differentiating healthy aging from dementia and studies detecting and quantifying lesions and imaging features associated with dementia and stroke.
2. Interpretation: Most of the studies assessed Alzheimer's disease (AD) and healthy controls from the AD Neuroimaging Initiative data set. Although accuracy was high when differentiating AD from healthy controls, performances were poorer when assessing more clinically relevant distinctions, such as classifying controls versus mild cognitive impairment versus AD or mild cognitive impairment converters versus nonconverters.
3. Future directions: Machine learning methods to predict risk of dementia do not seem ready for routine clinical use. More public, clinically relevant datasets, multisequence approaches, clinical variables, and multidisciplinary approaches need to be considered to ensure that machine learning methods are robust and reliable when applied to individual patients.

## Supplementary data

Supplementary Material

Supplementary Prospero

Supplementary Prisma
