# Supplementary material for: Machine learning of neuroimaging for assisted diagnosis of cognitive impairment and dementia: A systematic review
Source: Alzheimers Dement (Amst). 2018 Aug 11;10:519–35. doi: 10.1016/j.dadm.2018.07.004 (PMC6197752; doi:10.1016/j.dadm.2018.07.004)
Supplement: Supplementary Material [file mmc1.docx]

**Supplementary Methods, Tables and Figures**

**Methods**

**Table S1** Acronyms used in Tables listing study results.

**Table S2**. Details of reason for rejection and proportions

**Table S3**. Machine learning studies that classified AD or MCI

**Table S4.** Machine learning methods for classification of other types of dementia.

**Table S5**. Machine learning studies on lesion segmentation; top, white matter hyperintensities; bottom, ischaemic stroke lesions.

**Table S 6.** Machine learning for detection of specific (small) lesions; top, microbleeds (CMB); bottom, lacunes.

**Figure S1** Proportion of studies meeting relevant QUADAS criteria.

**Figure S2**. Forest plot of accuracy of studies for differentiating different cognitive states ordered by data source

**Figure S3**. Forest plot of accuracy of studies for differentiating different cognitive states ordered by machine learning method.

**Figure S4**. Forest plot of accuracy of studies for differentiating different cognitive states ordered by study size.

**References**

**Methods**

Calculation of the precision and recall for lesion identification tasks

Precision and Recall are defined as:

 Precision = TP/(TP + FP)

Recall = TP/(TP + FN)

where: TP = true positive (indicates accurate lesion identification)

TN = true negative (indicates correct rejection of non-lesion tissue)

FP = false positive (indicates identification of a lesion that is not there)

FN = false negative (indicates failure to identify a lesion that is present)

**Table S1**. Acronyms used in Tables listing study results.

| **Acronym** | **Definition** | | |  |
| --- | --- | --- | --- | --- |
| AD | Alzheimer’s Disease | | |  |
| AdaBoost | Adaptive Boosting | | |  |
| ADAS-Cog | Alzheimer’s Disease Assessment Scale - cognitive subtest | | |  |
| ADNI | Alzheimer’s Disease Neuroimaging Initiative | | |  |
| APOE3 | Apolipoprotein E | | |  |
| BoW | Bag of Words | | |  |
| CCA | Canonical Correlation Analysis | | |  |
| CDR-SB | Clinical Dementia Rating - Sum of Boxes | | |  |
| CMB | Cerebral Microbleeds | | |  |
| CSF | Cerebrospinal Fluid | | |  |
| DLB | Dementia with Lewy Bodies | | |  |
| DTI | Diffusion Tensor Imaging | | |  |
| DWT | Discrete Wavelet Transform | | |  |
| FA | Fractional Anisotropy | | |  |
| FAQ | Functional Activities Questionnaire | | |  |
| FDG | Fluorodeoxyglucose | | |  |
| FDR | Fisher Discriminant Ratio | | |  |
| FF-NN | Feed Forward Neural Network | | |  |
| FLAIR | Fluid Attenuated Inversion Recovery | | |  |
| fMRI | functional Magnetic Resonance Imaging | | |  |
| FS | Feature Selection | | |  |
| FTD | Frontotemporal Dementia | | |  |
| GM | Gray Matter | | |  |
| GRE | Gradient Recalled Echo | | |  |
| HC | Healthy Control | | |  |
| HMM | Hidden Markov Model | | |  |
| ICV | Intracranial Volume | | |  |
| kNN | k-Nearest Neighbours | | |  |
| LASSO | Least Absolute Shrinkage and Selection Operator | | |  |
| LBP | Local Binary Patterns | | |  |
| LDA | Linear Discriminant Analysis | | |  |
| MABMIS | Multi-Atlas based Multi-Image Segmentation | | |  |
| MCI | Mild Cognitive Impairment | | |  |
| MCIc | Mild Cognitive Impairment (converting) | | |  |
| MCIe | Mild Cognitive Impairment (early amnestic) | | |  |
| MCInc | Mild Cognitive Impairment (non-converting) | | |  |
| MD | Mean Diffusivity | | |  |
| MIL | Multiple Instance Learning | | |  |
| MKL | Multiple Kernel Learning | | |  |
| MMSE | Mini-Mental State Examination | |  |  |
| mRMR | | minimum Redundancy Maximum Relevance | | |
| MTI | | Magnetization Transfer Imaging | | |
| NN | | Neural Network | | |
| OASIS | | Open Access Series of Imaging Studies | | |
| OPLS | | Orthogonal Projections to Latent Structures | | |
| PCA | | Principal Component Analysis | | |
| PD | | Proton Density | | |
| PDF | | Probability Distribution Function | | |
| PESFAM | | Probabilistic Ensemble Simplified Fuzzy ARTMAP | | |
| PET | | Positron Emission Tomography | | |
| P-NN | | Probabilistic Neural Network | | |
| QDA | | Quadratic Discriminant Analysis | | |
| QDC | | Quadratic Discriminant Classifier | | |
| RAVENS | | Regional Analysis of Volumes Examined in Normalized Space | | |
| RAVLT | | Rey’s Auditory Verbal Learning Test | | |
| RBF | | Radial Basis Function | | |
| RFE | | Recursive Feature Elimination | | |
| ROI | | Region Of Interest | | |
| SAE | | Stacked Auto Encoders | | |
| SES | | Socioeconomic Status | | |
| SRC | | Sparse Representation Classification | | |
| SVD | | Small Vessel Disease | | |
| SVM | | Support Vector Machine | | |
| SWI | | Susceptibility-Weighted Imaging | | |
| TIA | | Transient Ischemic Attack | | |
| VBM | | Voxel-Based Morphometry | | |
| WM | | White Matter | | |
| WML | | White Matter Lesions |  | |

**Table S2**. Details of reason for rejection and proportions

| Rejection criteria | Number of rejected studies (%) |
| --- | --- |
| 1. Animals or ex-vivo. | 1 (0.2) |
| 1. Review, survey, collection. | 27 (5.3) |
| 1. Size of the dataset, number of observers. | 209 (40.7) |
| 1. Semi-automatic technique. | 61 (11.9) |
| 5. No structural imaging. | 25 (4.9) |
| 6. Pre-processing technique. | 12 (2.3) |
| 7. Healthy region parcellation. | 13 (2.5) |
| 8. Non-comparable results | 96 (18.7) |
| 9. ”Multiple” publications. | 63 (12.3) |
| 10. Non-reproducible | 7 (1.4) |

Table S3. Machine learning studies that classified AD or MCI and healthy controls

| Reference | Dataset | Classification | Image features | Additional imaging | Classifiers | Results |
| --- | --- | --- | --- | --- | --- | --- |
|  |  | tasks (n) | (FS and representation) | sequences and features |  |  |
| (Aggarwal, Rana et al. 2015) | OASIS | HC vs AD (99 / 99) | 3D-DWT (symmlet) of 7 ROI’s: hip- | n.a. | kNN | Sen = 0.789 / Spe = 0.810 |
|  |  |  | pocampus, amygdalae, ventricles, an- |  |  |  |
|  |  |  | terior and posterior cingulate (FS by |  |  |  |
|  |  |  | FDR and mRMR). |  |  |  |
| (Aguilar, Westman et al. 2013) | AddNeuroMed | HC vs AD (110 / 116) | 68 cortical thickness values and | Education. | SVM (non-lin) | Sen = 0.862 / Spe = 0.900 |
|  |  | MCInc vs MCIc (98 / 21) | 50 regional volumes obtained with | n.a. | OPLS | Sen = 0.810 / Spe = 0.684 |
|  |  |  | FreeSurfer. |  |  |  |
| (Ahmed, Mizotin et al. 2015) | ADNI | HC vs AD (162 / 137) | Circular harmonic features extracted | n.a. | SVM (RBF) | Sen = 0.791 / Spe = 0.882 |
|  |  | HC vs MCI (162 / 210) | from hippocampus and posterior cingu- |  |  | Sen = 0.626 / Spe = 0.748 |
|  |  | AD vs MCI (210 / 137) | late cortex (FS by PCA; BoW represen- |  |  | Sen = 0.490 / Spe = 0.752 |
|  |  |  | tation) |  |  |  |
| (Anagnostopoulos, Giannoukos et al. 2013) | AddNeuroMed | HC vs MCI vs AD | Cortical volume and thickness for spe- | T2w, demographics. | Ensemble of | Acc = 0.771 |
|  |  | (113 / 122 / 123) | cific ROI’s, manual volume measure- |  | FF-NN, SVM, |  |
|  |  |  | ment of the hippocampus. |  | PESFAM, |  |
|  |  |  |  |  | P-NN, kNN |  |
| (Archana and Ramakrishnan 2014) | OASIS | HC vs AD (92 / 45) | Voxel-wise texture features from struc- | n.a. | SVM | Sen = 0.877 / Spe = 0.849 |
|  |  | HC vs MCI (92 / 67) | ture tensor analysis (FS by FDR). |  |  | Sen = 0.764 / Spe = 0.783 |
|  |  | AD vs MCI (67 / 45) |  |  |  | Sen = 0.747 / Spe = 0.767 |
| (Babu, Suresh et al. 2013) | ADNI | HC vs MCIc (232 / 167) | Voxel-wise GM probability values from | n.a. | RBF-NN | Sen = 0.730 / Spe = 0.840 |
|  |  | MCInc vs MCIc (236 / 167) | VBM analysis (FS by t-test). |  |  | Sen = 0.880 / Spe = 0.890 |
| (Beheshti, Demirel et al. 2015) | ADNI | HC vs AD (130 / 130) | Voxel-wise GM probability values from | n.a. | SVM (RBF) | Sen = 0.908 / Spe = 0.908 |
|  |  |  | VBM analysis (FS based on PDF of |  |  |  |
|  |  |  | ROI’s). |  |  |  |
| (Casanova, Hsu et al. 2013) | ADNI | HC vs AD (188 / 171) | Voxel-wise intensities from GM, WM | n.a. | Regularized | Sen = 0.843 / Spe = 0.890 |
|  |  | HC vs MCInc (188 / 182) | and CSF maps. | n.a. | logistic | Sen = 0.586 / Spe = 0.681 |
|  |  | HC vs MCIc (188 / 153) |  | n.a. | regression | Sen = 0.740 / Spe = 0.881 |
|  |  | MCInc vs MCIc (182 / 153) |  | Cognitive scores. |  | Sen = 0.579 / Spe = 0.701 |
| (Chaddad, Desrosiers et al. 2016) | OASIS | HC vs AD (62 / 62) | 3D co-occurrence matrix. | n.a. | Random forest. | Sen = 0.742 / Spe = 0.759 |
| (Chen and Pham 2013) | OASIS | HC vs AD (75 / 75) | 2D regularity information from semi- | n.a. | HMM | Sen = 0.800 / Spe = 0.800 |
|  |  |  | variogram analysis of GM maps. |  |  |  |
| (Chen, Wei et al. 2015) | ADNI | MCInc vs MCIc (167 / 236) | GM volumes in 93 ROI’s (sparse repre- | n.a. | SRC | Sen = 0.581 / Spe = 0.763 |
|  |  |  | sentation). |  |  |  |
| (Chincarini, Bosco et al. 2011) | ADNI | HC vs AD (189 / 144) | Voxel intensities of filtered masks in 9 | n.a. | Random | Sen = 0.890 / Spe = 0.940 |
|  |  | HC vs MCIc (189 / 136) | ROI’s: hippocampi, amygdalae. middle |  | forest + SVM | Sen = 0.890 / Spe = 0.800 |
|  |  | MCInc vs MCIc (166 / 136) | and inf temp gyri, rolandic. |  |  | Sen = 0.720 / Spe = 0.650 |
| (Cho, Seong et al. 2012) | ADNI | HC vs AD (80 / 66) | Cortical thickness values (FS by PCA). | n.a. | LDA | Sen = 0.820 / Spe = 0.930 |
|  |  | HC vs MCIc (80 / 35) |  |  |  | Sen = 0.660 / Spe = 0.890 |
|  |  | MCInc vs MCIc (66 / 35) |  |  |  | Sen = 0.630 / Spe = 0.760 |
| (Costafreda, Dinov et al. 2011) | AddNeuroMed | MCInc vs MCIc (81 / 22) | Thickness values of hippocampi. | n.a. | SVM (RBF) | Sen = 0.770 / Spe = 0.800 |
| (Coupé, Fonov et al. 2015) | ADNI | MCInc vs MCIc (309 / 37) | SNIPE (Scoring by Nonlocal Image | n.a. | LDA | Sen = 0.649 / Spe = 0.735 |
|  |  |  | Patch Estimator) hippocampal fea- |  |  |  |
|  |  |  | tures. |  |  |  |
| (Cui, Wen et al. 2012) | Local | HC vs MCI (204 / 79) | 10 regional volumes from T1w, 58 WM | DTI. | SVM (RBF) | Sen = 0.520 / Spe = 0.784 |
|  |  |  | integrity features from DTI. |  |  |  |
| (Cuingnet, Gerardin et al. 2011) | ADNI | HC vs AD (80 / 66) | Voxel-wise GM probability values in | n.a. | SVM (linear) | en = 0.810 / Spe = 0.950 |
|  |  | HC vs MCIc (80 / 35) | ROI’s defined by different processing |  |  | Sen = 0.680 / Spe = 0.950 |
|  |  | MCInc vs MCIc (66 / 35) | pipelines. |  |  | Sen = 0.570 / Spe = 0.780 |
| (Cuingnet, Glaunes et al. 2013) | ADNI | HC vs AD (81 / 68) | GM, WM and CSF probability maps, | n.a. | SVM (non-lin) | Sen = 0.880 / Spe = 0.930 |
|  |  |  | cortical thickness values (FS by |  |  |  |
|  |  |  | anatomical and spatial priors in SVM). |  |  |  |
| (Davatzikos, Bhatt et al. 2011) | ADNI | MCInc vs MCIc (85 / 35) | Pattern of atrophy in GM and WM | CSF biomarkers. | SVM (non-lin) | Sen = 0.842 / Spe = 0.512 |
|  |  |  | maps. |  |  |  |
| (Ding, Zhang et al. 2015) | ADNI | HC vs AD (58 / 54) | 8 GM volumes in ROI’s, 220 texture | n.a. | SVM | Sen = 0.870 / Spe = 0.983 |
|  |  |  | features, 64 features from 2D multi- |  |  |  |
|  |  |  | scale Gabor filtering (FS by RFE). |  |  |  |
| (Dubey, Zhou et al. 2014) | ADNI | HC vs AD (191 / 138) | Cortical thickness values, volumes of | n.a. | Random forest | Sen = 0.826 / Spe = 0.906 |
|  |  | HC vs MCI (191 / 319) | cortical ROI’s, volumes of WM in |  | SVM | Sen = 0.793 / Spe = 0.493 |
|  |  | HC vs AD+MCIc (191 / 280) | ROI’s, total surface area of the cortex |  | SVM | Sen = 0.879 / Spe = 0.825 |
|  |  |  | (FS by sparse logistic regression). |  |  |  |
| (Dyrba, Ewers et al. 2012) | Local | HC vs AD (143 / 137) | GM probability map values (FS by | n.a. | SVM (RBF) | Sen = 0.874 / Spe = 0.912 |
|  |  |  | entropy-based information gain). |  |  |  |
| (Eskildsen, Coupé et al. 2013) | ADNI | HC vs AD (226 / 194) | Cortical thickness values (FS by t-test | Age. | LDA | Sen = 0.794 / Spe = 0.889 |
|  |  | MCInc vs MCIc (227 / 161) | and mRMR). |  |  | Sen = 0.658 / Spe = 0.683 |
| (Eskildsen, Coupé et al. 2015) | ADNI | MCInc vs MCIc (238 / 167) | L / R hippocampal grading, cortical | Age. | LDA | Sen = 0.696 / Spe = 0.736 |
|  |  |  | thickness values of 3 ROI’s (FS by mu- |  |  |  |
|  |  |  | tual information method). |  |  |  |
| (Filipovych, Davatzikos et al. 2011) | ADNI | HC vs AD (63 / 54) | GM RAVENS map (FS by RFE). | n.a. | SVM (linear, | Sen = 0.796 / Spe = 0.857 |
|  |  | MCInc vs MCIc (174 / 68) |  |  | semi-supervised) | Sen = 0.794 / Spe = 0.517 |
| (Granziera, Daducci et al. 2015) | Local | HC vs MCI (77 / 42) | Volume and mean intensity values from | MTI, T2*. | SVM | Sen = 0.600 / Spe = 0.830 |
|  |  |  | 7 ROI’s (WM and cortical GM, tha- |  |  |  |
|  |  |  | lamus, caudate, globus pallidus, puta- |  |  |  |
|  |  |  | men and hippocampus) and for WM |  |  |  |
|  |  |  | and GM of each lobe. |  |  |  |
| (Gray, Aljabar et al. 2013) | ADNI | HC vs MCI (35 / 75) | 83 ROI volumes from GM maps, voxel | PET, APOE3, | Random | Sen = 0.775 / Spe = 0.679 |
|  |  |  | intensities from PET. | CSF biomarkers. | forest |  |
| (Guerrero, Wolz et al. 2014) | ADNI | HC vs AD (175 / 106) | Voxel intensities (Laplacian eigenmaps | n.a. | SVM (linear) | Sen = 0.860 / Spe = 0.850 |
|  | ADNI-GO | MCInc vs MCIc (114 / 116) | representation after FS by Elastic Net |  |  | Sen = 0.750 / Spe = 0.670 |
|  |  | HC vs MCIc (175 / 116) | and manifold learning). |  |  | Sen = 0.860 / Spe = 0.760 |
|  |  | HC vs MCIe (134 / 229) |  |  |  | Sen = 0.610 / Spe = 0.690 |
| (Herrera, Rojas et al. 2013) | ADNI | HC vs AD (443 / 459) | 2D-DWT (Db4 and Haar) multi-scale | n.a. | SVM (RBF) | Sen = 0.963 / Spe = 0.961 |
|  |  | HC vs MCI vs AD (443 | features (FS by PCA and mutual infor- |  |  | Acc = 0.774 |
|  |  | / 448 / 459) | mation method). |  |  |  |
| (Hinrichs, Singh et al. 2009) | ADNI | HC vs AD (94 / 89) | GM probability maps (FS by t-test to | n.a. | Linear | Sen = 0.850 / Spe = 0.800 |
|  |  |  | select relevant voxels). |  | programming |  |
| (Hinrichs, Singh et al. 2011) | ADNI | HC vs AD (66 / 48) | GM probability maps (FS by t-test to | PET, APOE3, | MKL | Sen = 0.867 / Spe = 0.966 |
|  |  |  | select relevant voxels). | CSF biomarkers, |  |  |
|  |  |  |  | cognitive scores. |  |  |
| (Hor and Moradi 2016) | ADNI | HC vs MCIc (178 / 96) | Volume measurements of six ROI’s | n.a. | Random | Sen = 0.691 / Spe = 0.844 |
|  |  | HC vs MCInc (178 / 126) | (ventricles, hippocampus, whole-brain, | n.a. | forest | Sen = 0.734 / Spe = 0.863 |
|  |  | HC vs MCInc (18 / 144) | entorhinal, fusiform and mid-temporal) | PET. |  | Sen = 0.737 / Spe = 0.897 |
|  |  | MCInc vs MCIc (126 / 96) | and ICV from T1w, FDG and AV45 up- | n.a. |  | Sen = 0.819 / Spe = 0.750 |
|  |  | MCInc vs MCIc (144 / 27) | take values from PET (FS by informa- | PET. |  | Sen = 0.831 / Spe = 0.803 |
|  |  |  | tion gain). |  |  |  |
| (Hu, Wang et al. 2016) | ADNI | HC vs AD (228 / 188) | 3D-DWT (Gabor and Haar) multi-scale | n.a. | SVM (linear) | Sen = 0.846 / Spe = 0.855 |
|  |  | MCInc vs MCIc (62 / 71) | features from GM of map of hippocam- |  |  | Sen = 0.718 / Spe = 0.823 |
|  |  |  | pus. |  |  |  |
| (Illan, Garriz et al. 2014) | ADNI | HC vs AD (76 / 63) | Binary values of GM, WM maps in 6 | n.a. | SVM (ensemble) | Sen = 0.926 / Spe = 0.845 |
|  |  | HC vs MCIc (76 / 110) | ROI’s: parahippocampal gyrus, lingual |  |  | Sen = 0.773 / Spe = 0.845 |
|  |  |  | gyrus, hippocampus, frontal lobe, pre- |  |  |  |
|  |  |  | central gyrus, temporal lobe (Bayesian |  |  |  |
|  |  |  | network representation). |  |  |  |

| Reference | Dataset | Classification | Image features | Additional imaging | Classifiers | Results |
| --- | --- | --- | --- | --- | --- | --- |
|  |  | tasks (n) | (FS and representation) | sequences and features |  |  |
| (Jiang and Shi 2014) | ADNI | HC vs AD (52 / 51) | 83 ROI volumes from GM maps (FS by | n.a. | kNN | Sen = 0.920 / Spe = 0.904 |
|  |  |  | sparse kernel entropy component anal- |  |  |  |
|  |  |  | ysis). |  |  |  |
| (Jie, Zhang et al. 2014) | ADNI | HC vs AD (52 / 51) | GM volumes and PET intensity values | PET, | MKL | Sen = 0.947 / Spe = 0.958 |
|  |  | HC vs MCI (52 / 99) | in 93 ROI’s(FS by manifold regularized | CSF biomarkers. |  | Sen = 0.894 / Spe = 0.708 |
|  |  |  | multitask learning method). |  |  |  |
| (Khedher, Ramirez et al. 2015) | ADNI | HC vs AD (229 / 188) | Voxel intensities in GM and WM maps | n.a. | SVM (linear) | Sen = 0.913 / Spe = 0.851 |
|  |  | HC vs MCI (229 / 401) | (FS by partial least square). |  |  | Sen = 0.822 / Spe = 0.816 |
|  |  | MCI vs AD (401 / 188) |  |  |  | Sen = 0.870 / Spe = 0.838 |
| (Komlagan, Ta et al. 2014) | ADNI | MCInc vs MCIc (236 / 166) | SNIPE (Scoring by Nonlocal Image | n.a. | SVM (linear) | Sen = 0.615 / Spe = 0.856 |
|  |  |  | Patch Estimator) hippocampal features |  |  |  |
|  |  |  | (FS by sparse logistic regression). |  |  |  |
| (Korolev, Symonds et al. 2016) | ADNI | MCInc vs MCIc (139 / 120) | Cortical thickness, volumes, curvature | Risk factors, | MKL | Sen = 0.834 / Spe = 0.764 |
|  |  |  | and surface area of 180 ROI’s (FS by | cognitive scores, |  |  |
|  |  |  | joint mutual information criterion). | proteomic data. |  |  |
| (Krashenyi, Ramirez et al. 2016) | ADNI | HC vs AD (229 / 188) | Mean ROI intensity values of GM and | PET. | Fuzzy inference | Sen = 0.933 / Spe = 0.922 |
|  |  | HC vs MCI (229 / 401) | WM maps from T1w and mean inten- |  | system. | Sen = 0.759 / Spe = 0.861 |
|  |  | MCI vs AD (401 / 188) | sity from PET (FS by t-test). |  |  | Sen = 0.749 / Spe = 0.820 |
| (Lebedev, Westman et al. 2014) | ADNI | HC vs AD (75 / 35) | Volumes from 41 ROI’s and corti- | APOE3, | Random | Sen = 0.920 / Spe = 0.886 |
|  |  | MCInc vs MCIc (130 / 35) | cal thickness values (FS by PCA and | demographics. | forest. | Sen = 0.833 / Spe = 0.813 |
|  |  |  | RFE). |  |  |  |
| (Li, Wang et al. 2010) | OASIS | HC vs MCI (80 / 89) | GM values in 19 ROI’s (FS by t-test | MMSE. | SVM (RBF) | Sen = 0.919 / Spe = 0.880 |
|  |  |  | and feature ranking). |  |  |  |
| (Li, Liu et al. 2014) | ADNI | MCIc vs MCInc (161 / 132) | Cortical thickness values, volumes of | Demographics, | Random | Sen = 0.667 / Spe = 0.814 |
|  |  |  | cortical ROI’s, volumes of WM in | genetic data, | forest |  |
|  |  |  | ROI’s, total surface area of the cortex | cognitive scores, |  |  |
|  |  |  | (FS by hierarchical Lasso method). | lab tests. |  |  |
| (Li, Oishi et al. 2014) | ADNI | HC vs AD (142 / 80) | Voxel-wise combination of 2D-LBP | n.a. | SVM | Sen = 0.804 / Spe = 0.827 |
|  |  | MCInc vs MCIc (142 / 141) | from axial, coronal and sagittal orienta- |  |  | Sen = 0.615 / Spe = 0.635 |
|  |  |  | tions (FS by t-test and a priori knowl- |  |  |  |
|  |  |  | edge). |  |  |  |
| (Li, Yan et al. 2015) | ADNI | HC vs AD (60 / 60) | Volume and 15 texture features from 4 | n.a. | SVM (RBF) | Sen = 0.927 / Spe = 0.973 |
|  |  | HC vs MCI (60 / 60) | structures (GM, WM, CSF, hippocam- |  |  | Sen = 0.804 / Spe = 0.864 |
|  |  |  | pus) in L / R hemispheres (FS by chain- |  |  |  |
|  |  |  | like agent genetic algorithm). |  |  |  |
| (Liu, Suk et al. 2013) | ADNI | HC vs AD (198 / 198) | Volume and cortical thickness from 68 | n.a. | SVM | Sen = 0.894 / Spe = 0.950 |
|  |  | HC vs MCI (198 / 198) | ROI’s (sparse representation and high- |  | (multi-kernel) | Sen = 0.778 / Spe = 0.854 |
|  |  |  | order graph matching for FS). |  |  |  |
| (Liu, Tosun et al. 2013) | ADNI | HC vs MCIc (138 / 97) | Values of volume from 94 ROI’s and | n.a. | Logistic | Sen = 0.650 / Spe = 0.630 |
|  |  | HC vs MCInc (138 / 93) | cortical thickness from 68 ROI’s (repre- |  | regression | Sen = 0.810 / Spe = 0.820 |
|  |  | HC vs AD (138 / 86) | sentation by local linear embedding FS |  |  | Sen = 0.860 / Spe = 0.930 |
|  |  | MCInc vs MCIc (93 / 97) | by Elastic Net). |  |  | Sen = 0.800 / Spe = 0.560 |
|  |  | MCInc vs AD (93 / 86) |  |  |  | Sen = 0.770 / Spe = 0.730 |
|  |  | MCIc vs AD (97 / 86) |  |  |  | Sen = 0.560 / Spe = 0.610 |
| (Liu, Zhang et al. 2013) | ADNI | HC vs AD (229 / 198) | Voxel-wise GM probability values (reg- | n.a. | SVM (linear) | Sen = 0.853 / Spe = 0.943 |
|  |  | HC vs MCInc (229 / 236) | ularized tree-structured approach for |  |  | Sen = 0.801 / Spe = 0.922 |
|  |  | MCInc vs MCIc (236 / 167) | sparse learning). |  |  | Sen = 0.562 / Spe = 0.809 |
| (Liu, Zhou et al. 2014) | ADNI | HC vs AD (70 / 50) | 126 hippocampal shape features and | CSF biomarkers. | MKL | Sen = 0.933 / Spe = 0.875 |
|  |  |  | GM volumes from 100 ROI’s/ (FS by |  |  |  |
|  |  |  | LASSO). |  |  |  |
| (Liu, Liu et al. 2015) | ADNI | HC vs AD (204 / 180) | Values of volume from T1w and of | n.a. | Deep | Sen = 0.868 / Spe = 0.778 |
|  |  | HC vs MCI (204 / 374) | metabolic rate of glucose consumption | n.a. | learning | Sen = 0.495 / Spe = 0.843 |
|  |  | HC vs MCInc vs MCIc vs AD | from PET in 83 ROI’s (FS by Elastic | n.a. | (SAE) | Acc = 0.463 |
|  |  | (204 / 214 / 160 / 180) | Net). |  |  | Sen = 0.923 / Spe = 0.904 |
|  |  | HC vs AD (77 / 85) |  | PET |  | Sen = 0.600 / Spe = 0.923 |
|  |  | HC vs MCI (77 / 169) |  | PET |  | Acc = 0.538 |
|  |  | HC vs MCInc vs MCIc vs AD |  | PET |  |  |
|  |  | (77 / 102 / 67 / 85) |  |  |  |  |
| (Liu, Cai et al. 2016) | ADNI | HC vs MCI vs AD (77 / 169 / 85) | GM Volume, local gyrification index, | PET. | SVM | ACC = 0.6535 |
|  |  |  | convexity and solidity ratios from T1w, |  | (multi-kernel) |  |
|  |  |  | mean index, fuzzy index, 3 difference- |  |  |  |
|  |  |  | of-Gaussian features from PET in 83 |  |  |  |
|  |  |  | ROI’s. |  |  |  |
| (Liu, Zhang et al. 2016) | ADNI | HC vs AD (128 / 97) | ROI average values from different tem- | n.a. | SVM (ensemble) | Sen = 0.928 / Spe = 0.957 |
|  |  | MCInc vs MCInc (117 / 117) | plates of GM density maps (FS and en- |  |  | Sen = 0.860 / Spe = 0.784 |
|  |  |  | coding by subclass clustering). |  |  |  |
| (Luchtenberg, Simões et al. 2014) | OASIS | HC vs AD (66 / 70) | Dissimilarity matrix of voxel intensity | n.a. | kNN | Sen = 0.800 / Spe = 0.880 |
|  |  |  | histograms. |  |  |  |
| (Martinez-Torteya, Treviño et al. 2015) | ADNI | MCI vs AD (86 / 24) | GM volume in 90 ROI’s, cortical thick- | PET, CSF | LDA | Sen = 0.476 / Spe = 0.941 |
|  |  |  | ness in 139 ROI’s from T1w, metabolic | biomarkers, APOE3, |  |  |
|  |  |  | rate of glucose consumption from PET | plasma, biological |  |  |
|  |  |  | (FS by genetic models and Pearson cor- | samples. |  |  |
|  |  |  | relation coefficients). |  |  |  |
| (Martinez-Murcia, Gorriz et al. 2016) | ADNI | HC vs AD (180 / 180) | LBP features from GM and WM maps | n.a. | SVM (linear) | Sen = 0.899 / Spe = 0.919 |
|  |  |  | (2D representation of the brain and FS |  |  |  |
|  |  |  | by t-test). |  |  |  |
| (McEvoy, Fennema-Notestine et al. 2009) | ADNI | HC vs AD (139 / 84) | Morphometric measures from 58 ROI’s. | n.a. | LDA | Sen = 0.830 / Spe = 0.930 |
| (Moradi, Pepe et al. 2015) | ADNI | MCInc vs MCIc (100 / 164) | Voxel-wise GM density values (FS by | Age, RAVLT, | Random | Sen = 0.870 / Spe = 0.740 |
|  |  |  | regularized logistic regression). | ADAS-Cog, MMSE, | forest + |  |
|  |  |  |  | CDR-SB, FAQ. | SVM (RBF) |  |
| (Morgado and Silveira 2015) | ADNI | HC vs AD (75 / 59) | Voxel-wise GM density values (FS | n.a. | SVM | Sen = 0.869 / Spe = 0.872 |
|  |  | HC vs MCI (75 / 135) | by Minimal Neighborhood Redundancy |  |  | Sen = 0.688 / Spe = 0.670 |
|  |  |  | Maximal Relevance). |  |  |  |
| (Nho, Shen et al. 2010) | ADNI | HC vs AD (226 / 182) | GM density values from 86 ROI’s, cor- | APOE3, | SVM (RBF) | Sen = 0.850 / Spe = 0.948 |
|  |  | HC vs MCI (226 / 355) | tical thickness values from 56 ROI’s (FS | family history. |  | Sen = 0.694 / Spe = 0.698 |
|  |  |  | by SVM-RFE). |  |  |  |
| (Plocharski and Østergaard 2016) | ADNI | HC vs AD (96 / 109) | Depth, length, curvature and surface | n.a. | SVM (linear) | Sen = 0.900 / Spe = 0.867 |
|  |  |  | area of 24 sulci (FS by forward selec- |  |  |  |
|  |  |  | tion). |  |  |  |
| (Rao, Lee et al. 2011) | Local | HC vs AD (60 / 69) | Voxel-wise GM density values (FS by | n.a. | Logistic | Sen = 0.904 / Spe = 0.803 |
|  |  |  | spatially regularized formulation). |  | regression |  |
| (Rueda, Gonzalez et al. 2014) | OASIS | HC vs MCI + AD (98 / 100) | Voxel intensities (graph-based saliency | n.a. | MKL | Sen = 0.670 / Spe = 0.735 |
|  |  |  | map representation). |  |  |  |

| Reference | Dataset | Classification | Image features | Additional imaging | Classifiers | Results |
| --- | --- | --- | --- | --- | --- | --- |
|  |  | tasks (n) | (FS and representation) | sequences and features |  |  |
| (Savio and GrañA 2013) | OASIS | HC vs AD (316 / 100) | Voxel intensities (represented as the | n.a. | SVM (RBF) | Sen = 0.856 / Spe = 0.863 |
|  |  |  | trace of the Jacobian matrix from |  |  |  |
|  |  |  | tensor-based morphometry analysis. |  |  |  |
|  |  |  | FS by t-test). |  |  |  |
| (Schmitter, Roche et al. 2015) | ADNI | HC vs AD (276 / 221) | Volumes, obtained from FreeSurfer or | n.a. | SVM | Sen = 0.860 / Spe = 0.910 |
|  |  | HC vs MCI (276 / 401) | MorphoBox of: total GM, left and right |  |  | Sen = 0.690 / Spe = 0.830 |
|  |  | MCI vs AD (401 / 221) | temporal GM, left and right hippocam- |  |  | Sen = 0.690 / Spe = 0.670 |
|  |  | MCInc vs MCIc (103 / 137) | pus, total CSF, and lateral, 3 and 4 ven- |  |  | Sen = 0.750 / Spe = 0.660 |
|  |  |  | tricles. |  |  |  |
| (Schouten, Koini et al. 2016) | Local | HC vs AD (173 / 77) | GM density values in 110 ROI’s, WM | DTI, fMRI. | Regularized | Sen = 0.826 / Spe = 0.927 |
|  |  | HC vs AD mild (173 / 39) | density values in 20 ROI’s from T1w. |  | logistic | Sen = 0.721 / Spe = 0.935 |
|  |  | HC vs AD moderate | FA and MD values in 20 ROI’s. 2415 |  | regression | Sen = 0.813 / Spe = 0.956 |
|  |  | (173 / 38) | values of correlation from functional |  |  |  |
|  |  |  | connectivity analysis from fMRI (FS by |  |  |  |
|  |  |  | Elastic Net). |  |  |  |
| (Shi, Suk et al. 2014) | ADNI | HC vs AD (52 / 51) | GM volumes and PET intensity values | PET | SVM (linear) | Sen = 0.942 / Spe = 0.969 |
|  |  | HC vs MCI (52 / 99) | in 93 ROI’s (FS by Lasso). |  | SRC | Sen = 0.817 / Spe = 0.762 |
| (Singh, Fletcher et al. 2014) | ADNI | MCInc vs MCIc (73 / 54) | Anatomical shape variations with re- | PET, APOE3, | QDA | Sen = 0.942 / Spe = 0.969 |
|  |  |  | spect to atlas (FS by partial least | CSF biomarkers. |  | Sen = 0.817 / Spe = 0.762 |
|  |  |  | square model). |  |  |  |
| (Spulber, Simmons et al. 2013) | AddNeuroMed, | HC vs AD (52 / 51) | Volumes of 23 ROI’s and cortical thick- | PET | OPLS | Sen = 0.861 / Spe = 0.904 |
|  | ADNI | HC vs MCI (52 / 99) | ness values of 34 ROI’s. |  |  | Sen = 0.696 / Spe = 0.668 |
| (Suk, Lee et al. 2015) | ADNI | HC vs AD (52 / 51) | GM volumes and PET intensity val- | PET, | SVM | Sen = 0.920 / Spe = 0.980 |
|  |  | HC vs MCI (52 / 99) | ues in 93 ROI’s (FS by deep weighted | CSF biomarkers. |  | Sen = 0.939 / Spe = 0.908 |
|  |  | HC vs MCI vs AD(229 / | subclass-based sparse multi-task learn- |  |  | Acc = 0.577 |
|  |  | 403 / 198) | ing approach). |  |  |  |
|  |  | HC vs MCInc vs MCIc vs AD |  |  |  | Acc = 0.478 |
|  |  | (229 / 236 / 167 / 198) |  |  |  |  |
| (Tong, Wolz et al. 2014) | ADNI | HC vs AD (231 / 198) | Voxel intensity from a variable num- | n.a. | SVM | Sen = 0.849 / Spe = 0.926 |
|  |  | HC vs MCIc (231 / 167) | ber K of patches (MIL approach, FS by |  |  | Sen = 0.689 / Spe = 0.931 |
|  |  | MCInc vs MCIc (238 / 167) | Elastic Net). |  |  | Sen = 0.665 / Spe = 0.731 |
| (Varol, Gaonkar et al. 2012) | ADNI | HC vs AD (148 / 116) | Voxel-wise density values from GM, | n.a. | SVM (ensemble) | Sen = 0.862 / Spe = 0.897 |
|  |  |  | WM and ventricles maps (FS by t-test). |  |  |  |
| (Vemuri, Gunter et al. 2008) | ADNI | HC vs AD (50 / 50) | Voxel-wise density values from GM, | Demographics, | SVM (linear) | Sen = 0.860 / Spe = 0.920 |
|  |  |  | WM and CSF maps (FS by feature | APOE3. |  |  |
|  |  |  | ranking). |  |  |  |
| (Wachinger and Reuter 2016) | ADNI | HC vs MCI vs AD (129 / 122 / 103) | Cortical thickness values in 70 ROI’s, | n.a. | Multinomial | Acc = 0.590 |
|  |  |  | volumes of 39 ROI’s and 58 shape fea- |  | regression |  |
|  |  |  | tures. (FS by Elastic Net). |  |  |  |
| (Wang, Jia et al. 2012) | ADNI | HC vs AD (229 / 199) | GM, WM CSF values in 54 ROI’s ob- | n.a. | SVM (linear) | Sen = 0.861 / Spe = 0.918 |
|  |  | MCI vs AD (404 / 199) | tained from MABMIS pipeline (FS by |  |  | Sen = 0.403 / Spe = 0.870 |
|  |  | HC vs MCI (229 / 404) | t-test). |  |  | Sen = 0.794 / Spe = 0.881 |
|  |  | MCInc vs MCIc (236 / 168) |  |  |  | Sen = 0.667 / Spe = 0.723 |
| (Wang, Du et al. 2015) | ADNI | HC vs MCI (52 / 99) | GM volumes and PET intensity values | PET, | SVM (linear) | Sen = 0.827 / Spe = 0.473 |
|  |  |  | in 93 ROI’s (FS by PCA). | CSF biomarkers. |  |  |
| (Wee, Yap et al. 2012) | ADNI | HC vs AD (200 / 198) | Cortical thickness, GM and WM vol- | n.a. | SVM (RBF, | Sen = 0.904 / Spe = 0.943 |
|  |  | HC vs MCI (200 / 200) | umes in 68 ROI’s. Correlative features |  | multi-kernel) | Sen = 0.836 / Spe = 0.840 |
|  |  | MCI vs AD (200 / 198) | between pairs of ROI’s (FS by t-test, |  |  | Sen = 0.780 / Spe = 0.805 |
|  |  | MCInc vs MCIc (111 / 89) | mRMR and SVM-RFE). |  |  | Sen = 0.635 / Spe = 0.844 |
| (Wei, Li et al. 2016) | ADNI | MCInc vs MCIc (83 / 76) | Cortical thickness, volume, and cortical | n.a. | SVM (RBF) | Sen = 0.684 / Spe = 0.759 |
|  |  |  | surface area in 68 ROI’s. 136 nodal fea- |  |  |  |
|  |  |  | tures from the thickness network(FS by |  |  |  |
|  |  |  | regularized sparse linear regression). |  |  |  |
| (Westman, Simmons et al. 2011) | AddNeuroMed, | HC vs AD (335 / 295) | Cortical thicknessin in 57 selected | n.a. | OPLS | Sen = 0.834 / Spe = 0.878 |
|  | ADNI | MCInc vs MCIc (353 / 84) | ROI’s and volumes of 23 ROI’s. |  |  | Sen = 0.714 / Spe = 0.601 |
| (Wolz, Julkunen et al. 2011) | AddNeuroMed, | HC vs AD (231 / 198) | Hippocampal volume, cortical thickness | n.a. | LDA | Sen = 0.930 / Spe = 0.850 |
|  | ADNI | HC vs MCIc (231 / 167) | from different ROI’s, 84 tensor-based |  |  | Sen = 0.860 / Spe = 0.820 |
|  |  | MCInc vs MCIc (238 / 167) | morphometry and 20 manifold learning |  |  | Sen = 0.670 / Spe = 0.690 |
|  |  |  | features (FS by t-test). |  |  |  |
| (Xie, Cui et al. 2015) | Local | HC vs MCI (64 / 64) | Voxel-wise value of GM from T1w and | DTI. | SVM (linear, | Sen = 0.786 / Spe = 0.888 |
|  |  |  | FA and MD from DTI (FS by t-test). |  | ensemble) |  |
| (Xu, Wu et al. 2015) | ADNI | HC vs AD (117 / 113) | GM volumes from T1w and uptake val- | PET (FDG and | SRC | Sen = 0.956 / Spe = 0.940 |
|  |  | HC vs MCI (117 / 110) | ues from PET in 90 ROI’s (FS by t- | Florbetapir). |  | Sen = 0.664 / Spe = 0.821 |
|  |  | MCInc vs MCIc (83 / 27) | test). |  |  | Sen = 0.741 / Spe = 0.815 |
| (Yang, Li et al. 2014) | ADNI | HC vs AD (150 / 70) | Voxel-wise values from GM map (ICA | MMSE, | SVM | Sen = 0.992 / Spe = 0.962 |
|  |  | HC vs MCI (150 / 98) | decomposition and FS by t-test). | GDTOTAL, |  | Sen = 0.860 / Spe = 0.896 |
|  |  |  |  | HMSCORE. |  |  |
| (Ye, Pohl et al. 2011) | ADNI | MCInc vs MCIc (169 / 68) | GM RAVENS map (graph representa- | n.a. | SVM (linear | Sen = 0.941 / Spe = 0.408 |
|  |  |  | tion and FS by ISOMAP). |  | Laplacian, |  |
|  |  |  |  |  | semi-supervised) |  |
| (Ye, Zu et al. 2015) | ADNI | HC vs AD (52 / 51) | GM volumes and PET intensity val- | PET. | SVM (linear, | Sen = 0.947 / Spe = 0.971 |
|  |  | HC vs MCI (52 / 99) | ues in 93 ROI’s (FS by discriminative |  | multi-kernel) | Sen = 0.877 / Spe = 0.715 |
|  |  |  | multi-task approach). |  |  |  |
| (Young, Modat et al. 2013) | ADNI | MCInc vs MCIc (96 / 47) | GM volumes and PET intensity values | PET, APOE3. | Gaussian | Sen = 0.787 / Spe = 0.656 |
|  |  |  | in 920 ROI’s. |  | process |  |
| (Zhang, Wang et al. 2011) | ADNI | HC vs AD (52 / 51) | GM volumes and PET intensity values | PET, | SMV (linear, | Sen = 0.930 / Spe = 0.933 |
|  |  | HC vs MCI (52 / 99) | in 93 ROI’s. | CSF biomarkers. | multi-kernel) | Sen = 0.818 / Spe = 0.660 |
| (Zhang, Wang et al. 2015) | OASIS | HC vs MCI vs AD | 3D-DWT decomposition features, ICV, | Demographics, | SMV (RBF) | Acc = 0.815 |
|  |  | (97 / 57 / 24) | atlas scaling factor, normalized brain | Education, |  |  |
|  |  |  | volume (FS by PCA.) | SES, MMSE. |  |  |
| (Zhang and Wang 2015) | OASIS | HC vs AD (98 / 28) | Voxel-wise displacement field values | PET, APOE3. | SVM (twin) | Sen = 0.906 / Spe = 0.934 |
|  |  |  | (direction and magnitude) of key T1w |  |  |  |
|  |  |  | slices. (FS by PCA.) |  |  |  |
| (Zhang, Stonnington et al. 2016) | ADNI | HC vs AD (228 / 194) | Hippocampal surface tensor-based mor- | n.a. | Adaboost | Sen = 0.830 / Spe = 0.780 |
|  |  | MCInc vs MCIc (246 / 142) | phometry features and radial distance |  |  | Sen = 0.820 / Spe = 0.760 |
|  |  |  | (FS by sparse coding) |  |  |  |
| (Zheng, Yao et al. 2015) | ADNI | HC vs AD (189 / 163) | Cortical thickness in 78 ROI’s (cor- | APOE3. | SVM (RBF) | Sen = 0.899 / Spe = 0.943 |
|  |  | HC vs MCI (189 / 198) | relation matrix representation, FS by |  |  | Sen = 0.878 / Spe = 0.858 |
|  |  | MCI vs AD (198 / 163) | mRMR and SVM-RFE). |  |  | Sen = 0.806 / Spe = 0.899 |
|  |  | MCInc vs MCIc (94 / 104) |  |  |  | Sen = 0.789 / Spe = 0.799 |
| (Zheng, Shi et al. 2016) | ADNI | HC vs AD (52 / 51) | GM volumes and PET intensity values | PET. | SVM (linear) | Sen = 0.973 / Spe = 0.983 |
|  |  |  | in 93 ROI’s (high-level representation |  |  |  |
|  |  |  | from multi-modality stacked deep poly- |  |  |  |
|  |  |  | nomial network) |  |  |  |
| (Zhou, Goryawala et al. 2014) | ADNI | HC vs AD (127 / 59) | 41 regional and 10 morphometric vol- | MMSE. | SVM (RBF) | Sen = 0.840 / Spe = 0.961 |
|  |  | HC vs MCIc (127 / 67) | umes (FS by t-test). |  |  | Sen = 0.611 / Spe = 0.834 |
|  |  | HC vs MCInc (127 / 56) |  |  |  | Sen = 0.552 / Spe = 0.823 |
| (Zhu, Suk et al. 2014) | ADNI | HC vs AD (52 / 51) | GM volumes and PET intensity values | n.a. | SVM | Sen = 0.886 / Spe = 0.978 |
|  |  | HC vs MCI (52 / 99) | in 93 ROI’s (FS by regularized least |  |  | Sen = 0.948 / Spe = 0.569 |
|  |  |  | square regression). |  |  |  |
| (Zhu and Shi 2014) | ADNI | HC vs AD (52 / 51) | GM volumes in 93 ROI’s (co-training | n.a. | SVM (linear) | Sen = 0.869 / Spe = 0.904 |
|  |  |  | semi-supervised learning approach). |  |  |  |
| (Zhu, Shi et al. 2014) | ADNI | HC vs AD (52 / 51) | GM volumes and PET intensity values | n.a. | SVM (linear) | Sen = 0.952 / Spe = 0.907 |
|  |  |  | in 93 ROI’s (FS by Hessian regulariza- |  |  |  |
|  |  |  | tion semi-supervised approach). |  |  |  |
| (Zhu, Suk et al. 2015) | ADNI | HC vs MCI vs AD | GM volumes and PET intensity values | PET. | SMV | Acc = 0.729 |
|  |  | (52 / 99 / 51) | in 93 ROI’s (CCA representation and |  |  |  |
|  |  | HC vs MCInc vs MCIc vs AD | FS by multi-task learning). |  |  | Acc = 0.619 |
|  |  | (52 / 56 / 43 / 51) |  |  |  |  |

Table S4. Machine learning methods for classification of other types of dementia.

| Reference | Dataset | | Validation | | Tasks | | Imaging | | Imaging features | | Classifiers | | Acc | |  |
| --- | --- | --- | --- | --- | --- | --- | --- | --- | --- | --- | --- | --- | --- | --- | --- |
|  | (population) | | set size | |  | | sequences | | (FS and representation) | |  | |  | |  |
| (Chen, Tong et al. 2015) | Local | | 350 / 240 | | HC vs SVD | | CT | | Voxel intensities in ROI’s from WML-based | | MIL | | 0.75 | |  |
|  | (Stroke) | |  | |  | |  | | atlas. | |  | |  | |  |
| (Koikkalainen, Rhodius-Meester et al. 2016) | Local | | 118 / 223 / 92 | | HC vs AD vs FTD | | T1w, FLAIR. | | Volumes of 142 ROI’s, values of TBM and | | Multimodal | | 0.706 | |  |
|  | (Dementia) | | / 47 / 24 | | vs DLB vs SVD | |  | | VBM in 140 ROI’s, 20 manifold learning fea- | | statistical | |  | |  |
|  |  | |  | |  | |  | | tures, 8 ROI-based gradings and 1 vascular | | approach. | |  | |  |
|  |  | |  | |  | |  | | burden measure. | |  | |  | |  |
| (Oppedal, Eftestøl et al. 2015) | Local | | 36 / 57 / 16 | | HC vs AD vs LBD | | T1w, FLAIR. | | Voxel-wise 2D-LBP and contrast features from | | Random | | 0.87 | |  |
|  | (Dementia) | |  | |  | |  | | WM amd WML regions in T1w and FLAIR | | forest. | |  | |  |
|  |  | |  | |  | |  | | (FS by best first approach). | |  | |  | |  |
| (Vemuri, Simon et al. 2011) | Local | | 48 / 20 / 47 | | AD vs FTD vs LBD | | T1w. | | GM volumes in 91 ROI’s (FS by LDA). | | k-means | | 0.867 | |  |
|  | (Dementia) | |  | |  | |  | |  | |  | |  | |  |
| (Wang, Redmond et al. 2016) | Local | | 54 / 55 | | AD vs FTD | | T1w. | | 17 neurophysiological features and GM vol- | | Naive | | 0.6147 | |  |
|  | (Dementia) | | 57 / 54 / 55 | | HC vs AD vs FTD | |  | | umes of 8 ROI’ss (amygdala, hippocampus, | | Bayes | | 0.6747 | |  |
|  |  | |  | |  | |  | | medial temporal lobe, temporal pole, dorsolat- | |  | |  | |  |
|  |  | |  | |  | |  | | eral prefrontal cortex, ventromedial prefrontal | |  | |  | |  |
|  |  | |  | |  | |  | | cortex, striatum and insula (FS by best first | |  | |  | |  |
|  | |  | |  | |  | |  | | approach). | |  | |  | |

**Table S5:** Machine learning studies on lesion segmentation; top, white matter hyperintensities; bottom, ischaemic stroke lesions. DC = DICE coefficient where a value close to 1 indicates perfect match of the test segmentation with the reference standard and 0 indicates no overlap.

| Reference | Dataset | Validation | Target | Imaging | Imaging features | Classifiers | DC |
| --- | --- | --- | --- | --- | --- | --- | --- |
|  | (population) | set size |  | sequences | (FS and representation) |  |  |
| (Fiot, Cohen et al. 2013) | Local | 125 | WMH | T1w, T2w, | Neighbourhood voxel intensities and pyra- | SVM (RBF) | 0.69 |
|  | (Ageing) |  |  | FLAIR, PD. | midal features (Gaussian kernels) from each |  |  |
|  |  |  |  |  | modality in ROI’s. |  |  |
| (Ithapu, Singh et al. 2014) | Local | 38 | WMH | T1w, FLAIR. | Voxel intensities and textons in ROI’s. | Random | 0.67 |
|  | (AD and ageing) |  |  |  |  | forest |  |
| (Erus, Zacharaki et al. 2014) | Local | 80 | WMH | FLAIR. | Voxel-wise intensity values of abnormality | Iterative | 0.59 |
|  | (Diabetic |  |  |  | map in ROI’s (FS by wavelet-based approach). | wavelet-based |  |
|  | and ageing) |  |  |  |  | PCA model. |  |
| (Griffanti, Zamboni et al. 2016) | Local | 82 | WMH | FLAIR. | Spatially weighted voxel-wise intensity values, | kNN | 0.52 |
|  | (TIA or minor stroke, |  |  |  | patch average intensity. |  |  |
|  | no lacunar infarcts) |  |  |  |  |  |  |
| (Vos, Biesbroek et al. 2013) | Local | 30 | Stroke | CT. | Location and volume of lesion, voxel intensi- | Random | 0.74 |
|  | (Stroke) |  |  |  | ties and likelihood of belonging to a lesion in | forest |  |
|  |  |  |  |  | lesion and mirrored region (FS by first best |  |  |
|  |  |  |  |  | search approach). |  |  |
| (Guo, Fridriksson et al. 2015) | Local | 60 | Stroke | T1w. | Voxel-wise 0-, 1-, 2-order statistical features | SVM | 0.73 |
|  | (Stroke) |  |  |  | from T1w, GM, WM, CSF and lesion proba- | (linear, |  |
|  |  |  |  |  | bility map. | ensemble) |  |
| (Maier, Schröder et al. 2015) | Local | 35 | Stroke | FLAIR. | Voxel intensity and location, weighted mean | Random | 0.67 |
|  | (spatial neglect) |  |  |  | and histogram in voxel neighbourhood. | forest |  |

**Table S 6.** Machine learning for detection of specific (small) lesions; top, microbleeds (CMB); bottom, lacunes.

| Reference | Dataset | Validation | Target | Imaging | Imaging features | Classifiers | Pre | Rec |
| --- | --- | --- | --- | --- | --- | --- | --- | --- |
|  | (population) | set size |  | sequences | (FS and representation) |  |  |  |
| (Ghafaryasl, van der Lijn et al. 2012) | Local | 81 | CMB | T2*, GRE. | Intensity, size and shape features from candi- | Parzen, QDC | 0.352 | 0.99 |
|  | (Ageing) |  |  |  | date ROI’s in T2*. Intensity in GRE (FS by |  |  |  |
|  |  |  |  |  | feed-forward approach). |  |  |  |
| (Dou, Chen et al. 2016) | Local | 50 | CMB | SWI | 3D patches of SWI used as input. | 3D conv-NN | 0.443 | 0.93 |
|  | (Stroke and ageing) |  |  |  |  |  |  |  |
| (Fazlollahi, Meriaudeau et al. 2015) | Local | 66 | CMB | SWI | 3D Radon- and Hessian-based shape features | Random | 0.101 | 0.87 |
|  | (Diabetic |  |  |  | from candidate ROI’s. | forest (cascade) |  |  |
|  | and ageing) |  |  |  |  |  |  |  |
| (Uchiyama, Abe et al. 2014) | Local | 132 | Stroke | T1w, T2w | Location, intensity differences in T1w and | SVM | 0.154 | 0.97 |
|  | (lacunar infarcts) |  |  |  | T2w, multi-scale nodular and linear compo- |  |  |  |
|  |  |  |  |  | nent (FS by PCA). |  |  |  |


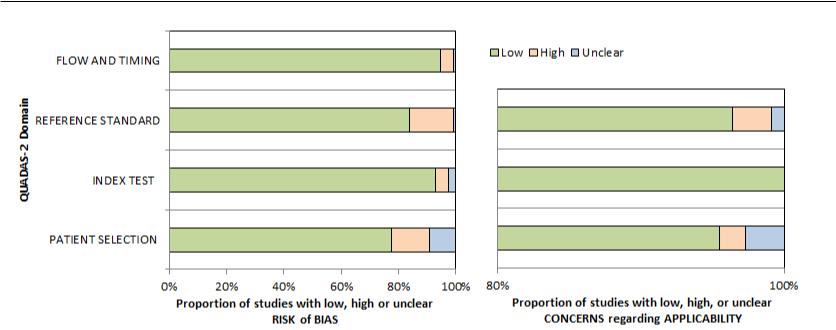
Figure S1 QUADAS-2 charts of the studies included in the review

Figure S2 – Forest plot of accuracy of studies for differentiating different cognitive states ordered by data source, 1^st^ page.


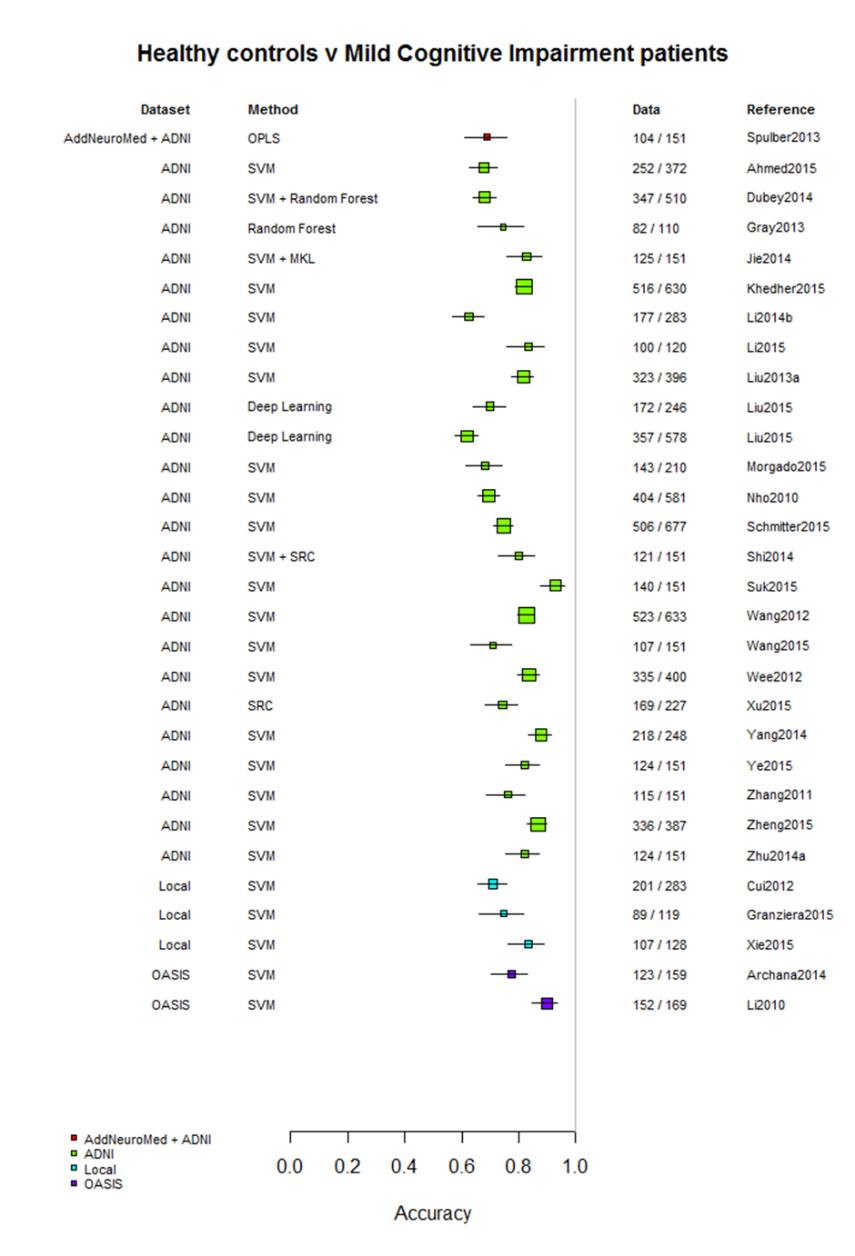

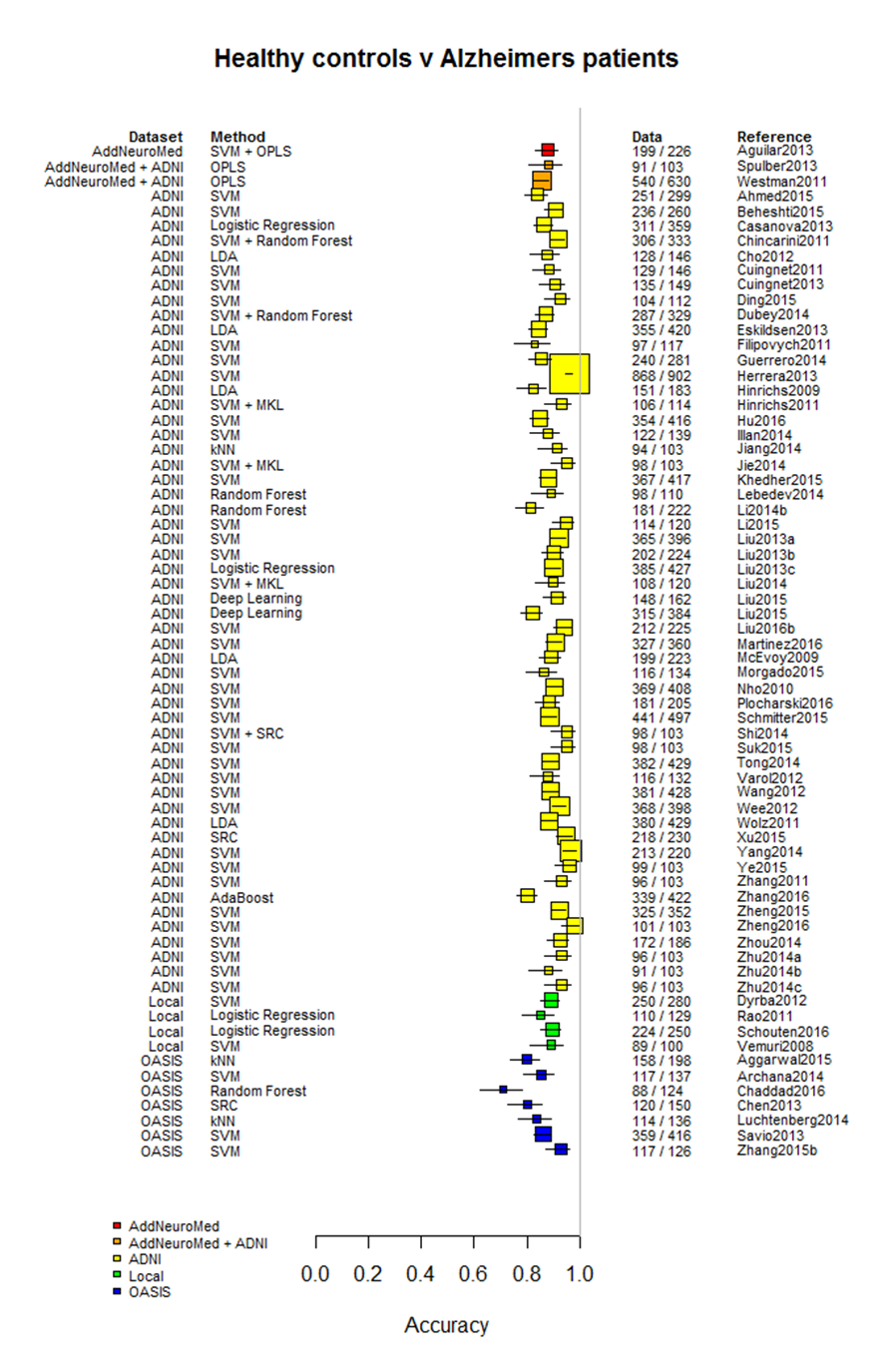


Figure S2 continued– Forest plot of accuracy of studies for differentiating different cognitive states ordered by data source, 2^nd^ page.


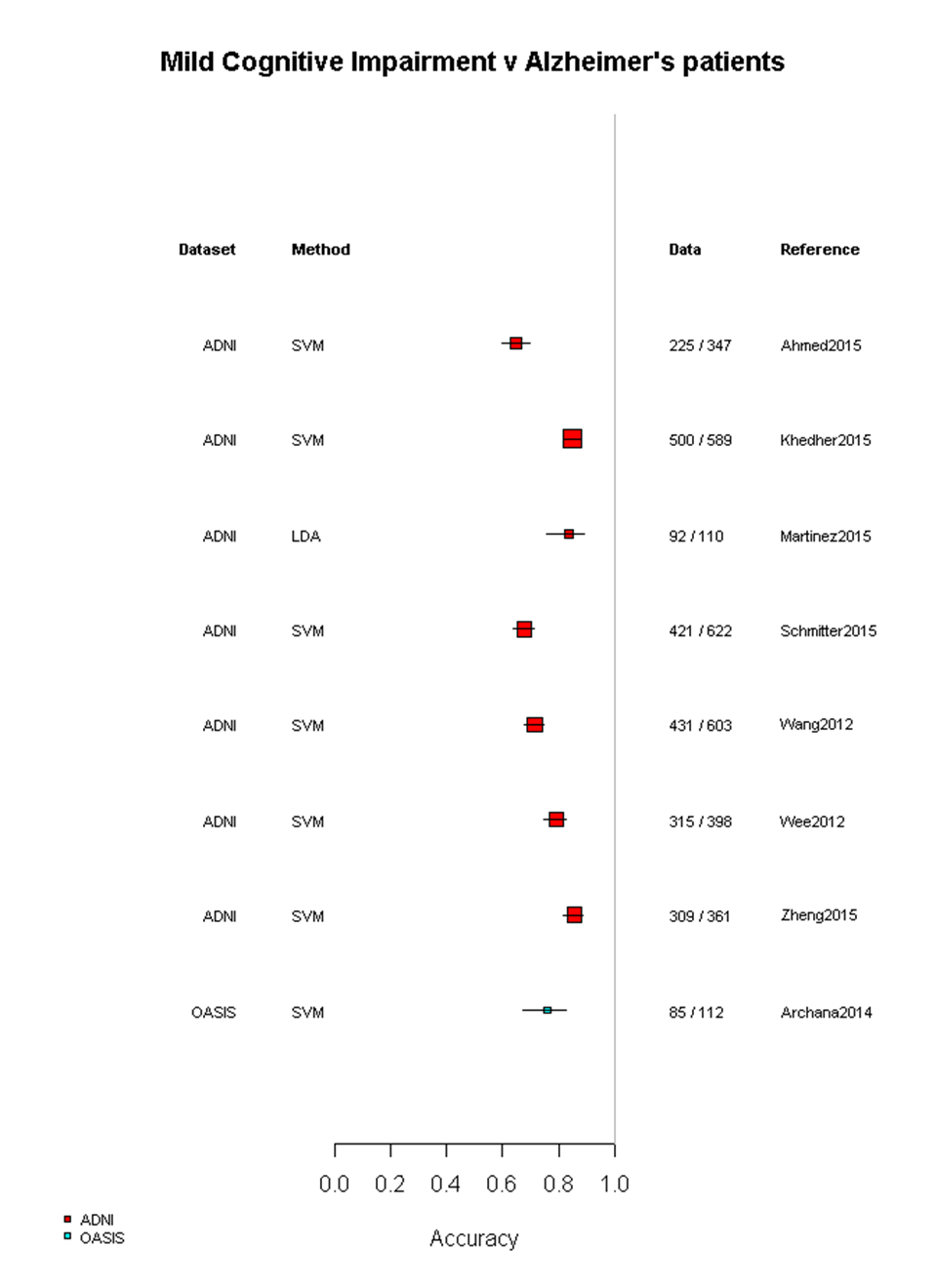

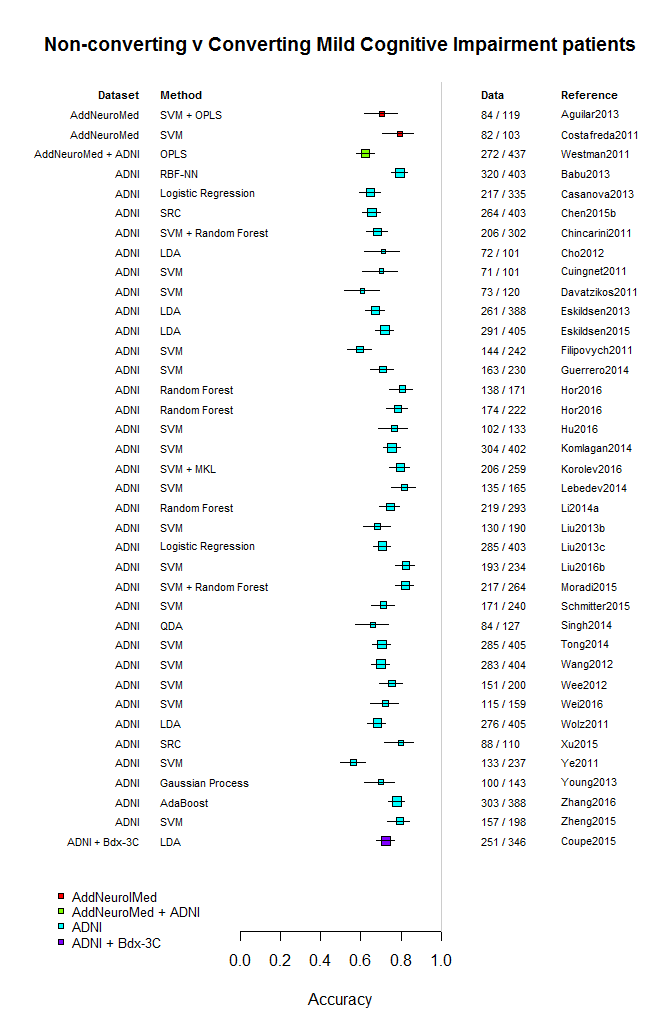


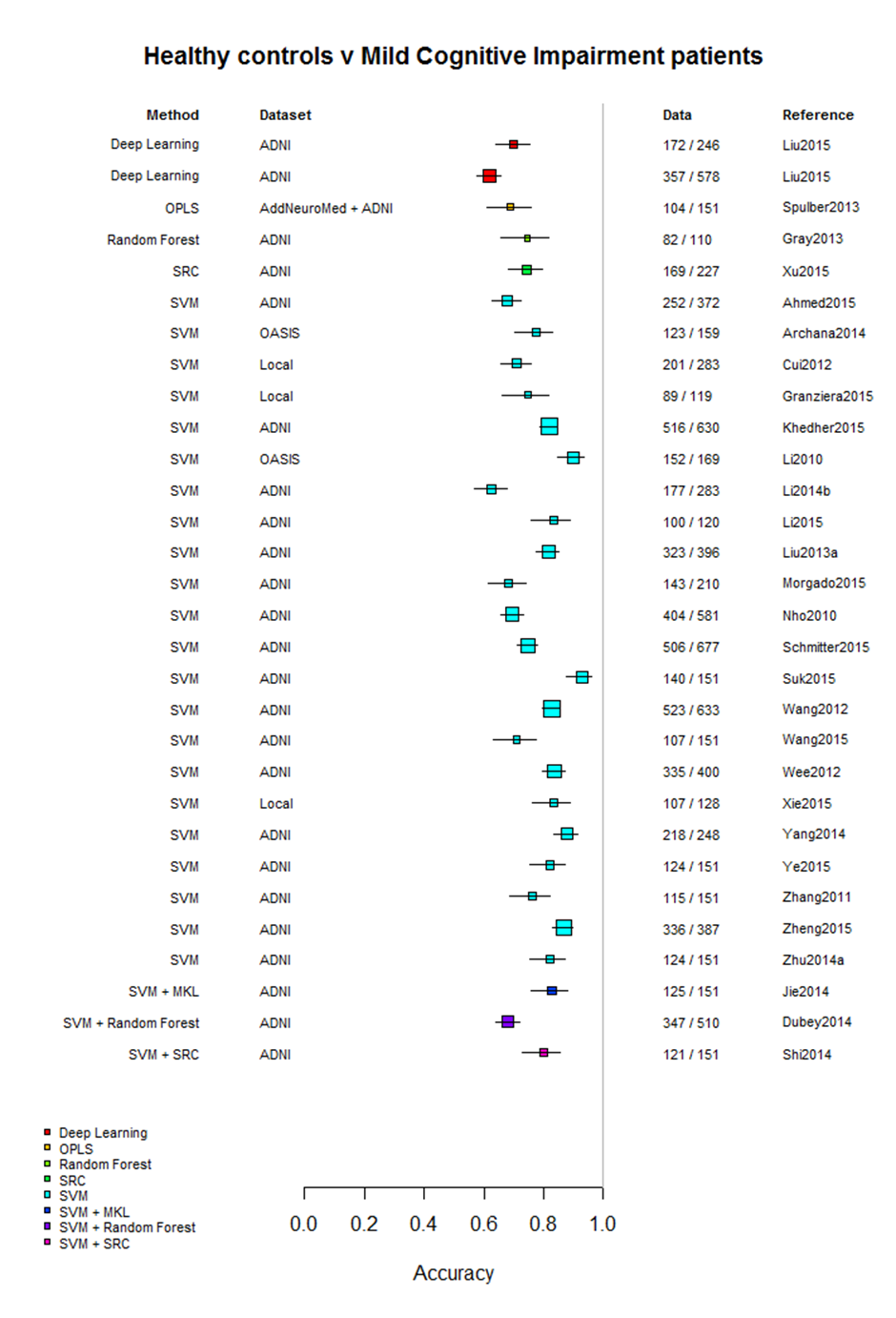

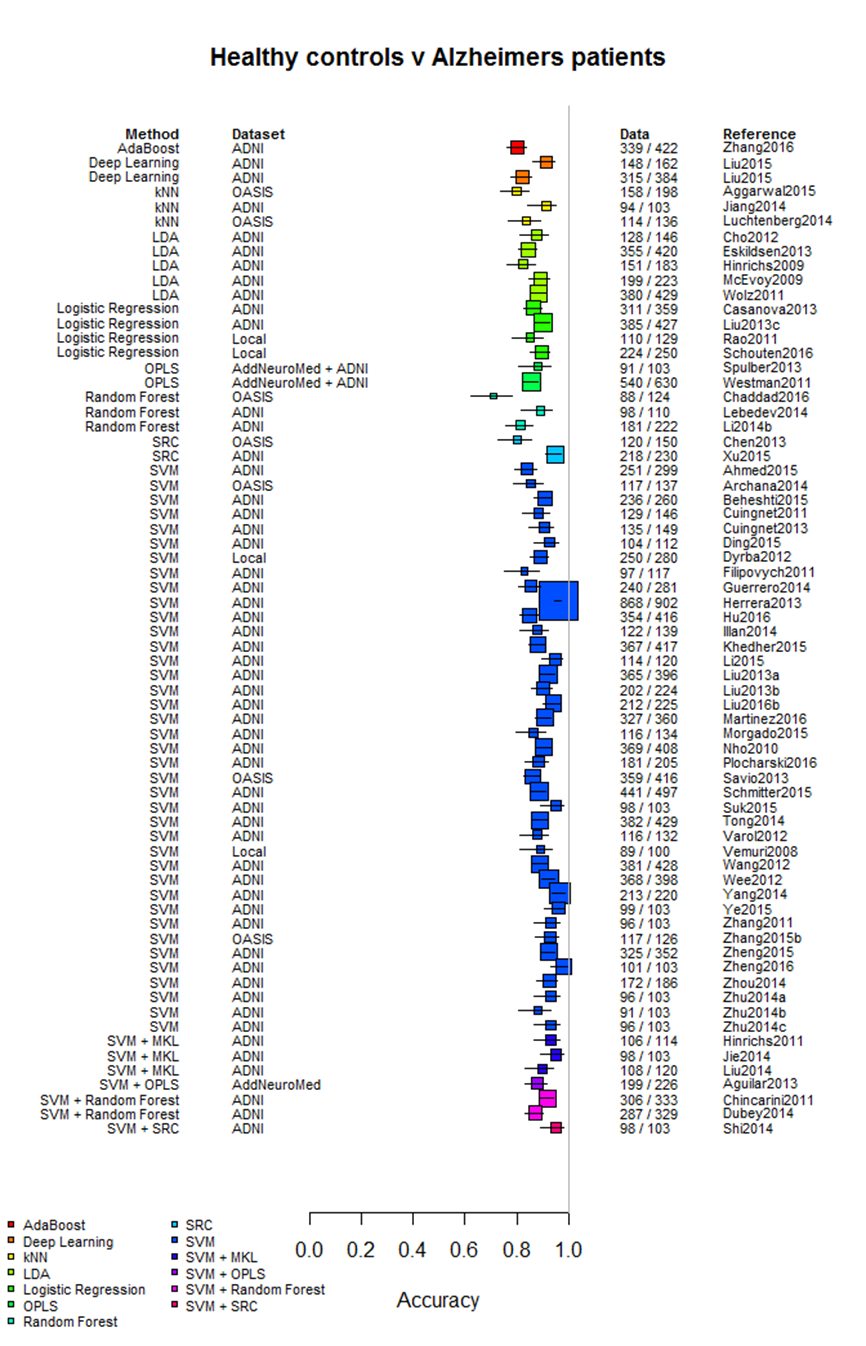
Figure S3 – Forest plot of accuracy of studies for differentiating different cognitive states ordered by machine learning method, 1^st^ page.

Figure S3 cont– Forest plot of accuracy of studies for differentiating different cognitive states ordered by machine learning method, 2^nd^ page


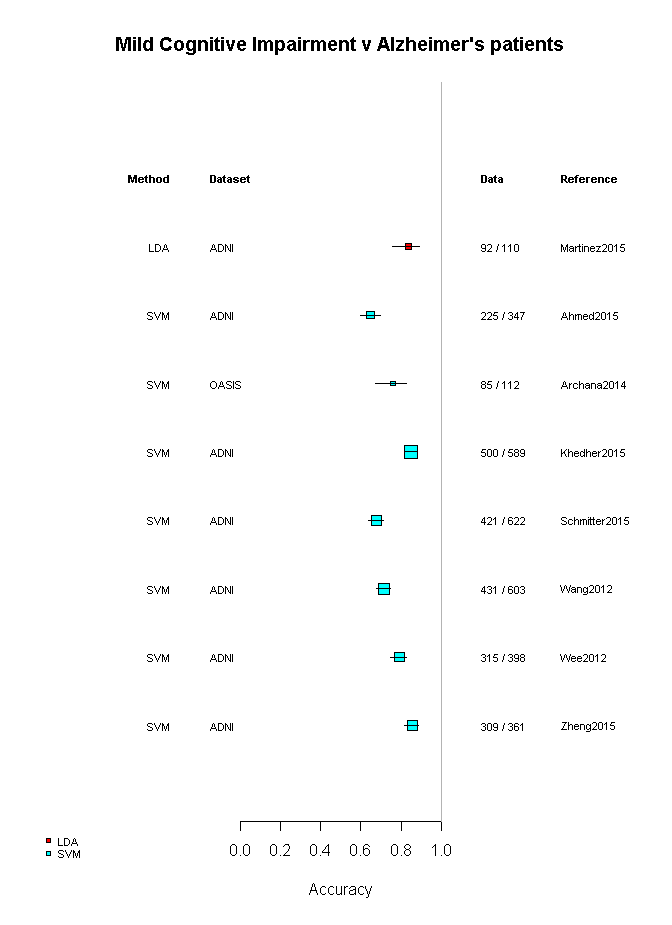

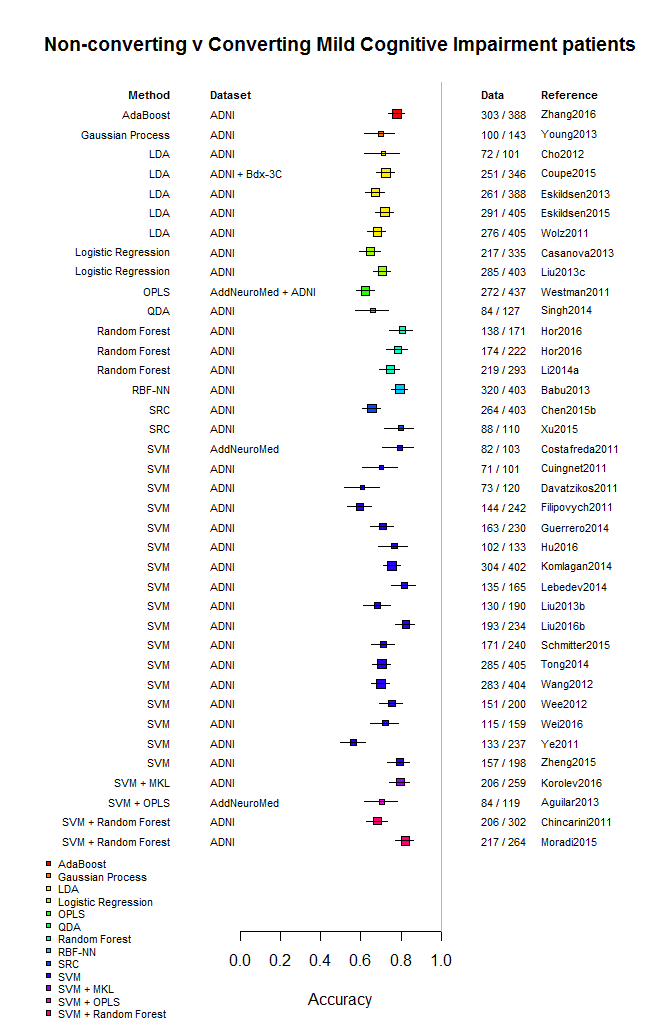


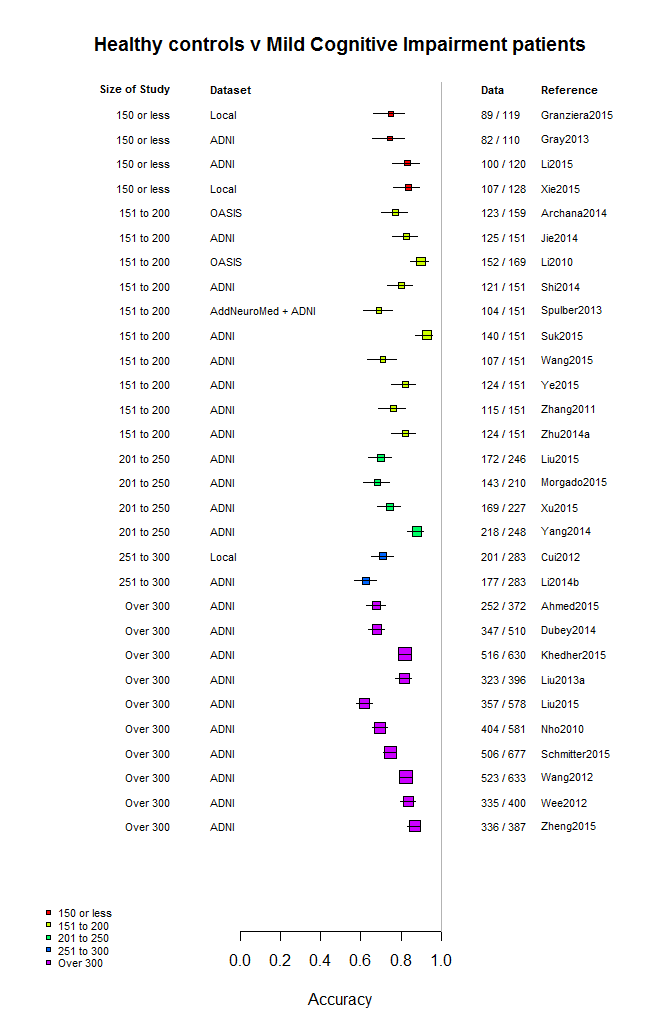

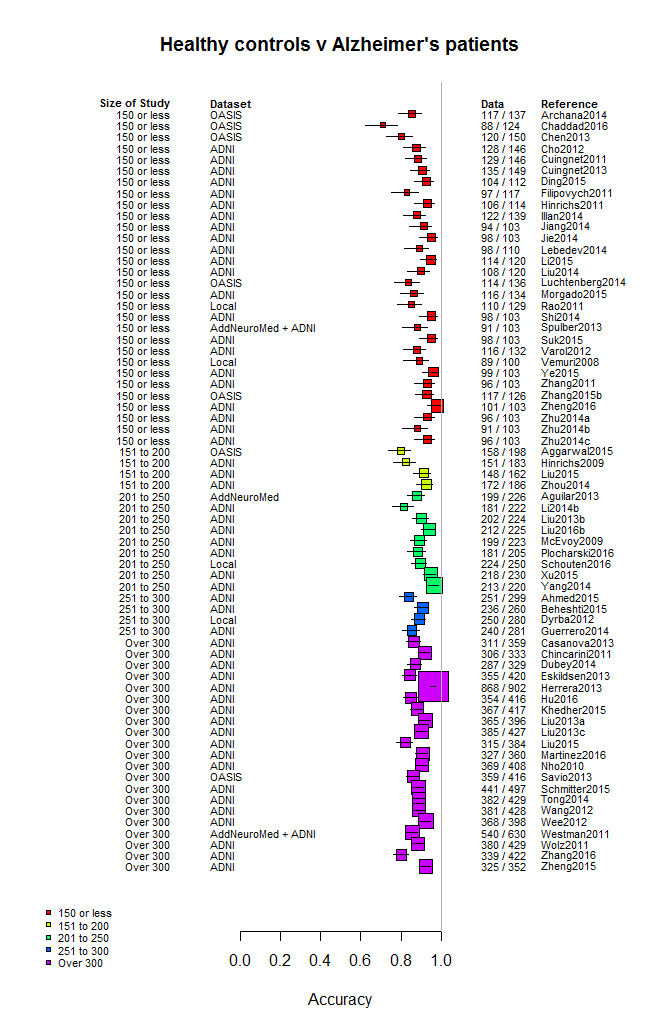
Figure S4 – Forest plot of accuracy of studies for differentiating different cognitive states ordered by study size, 1st page.


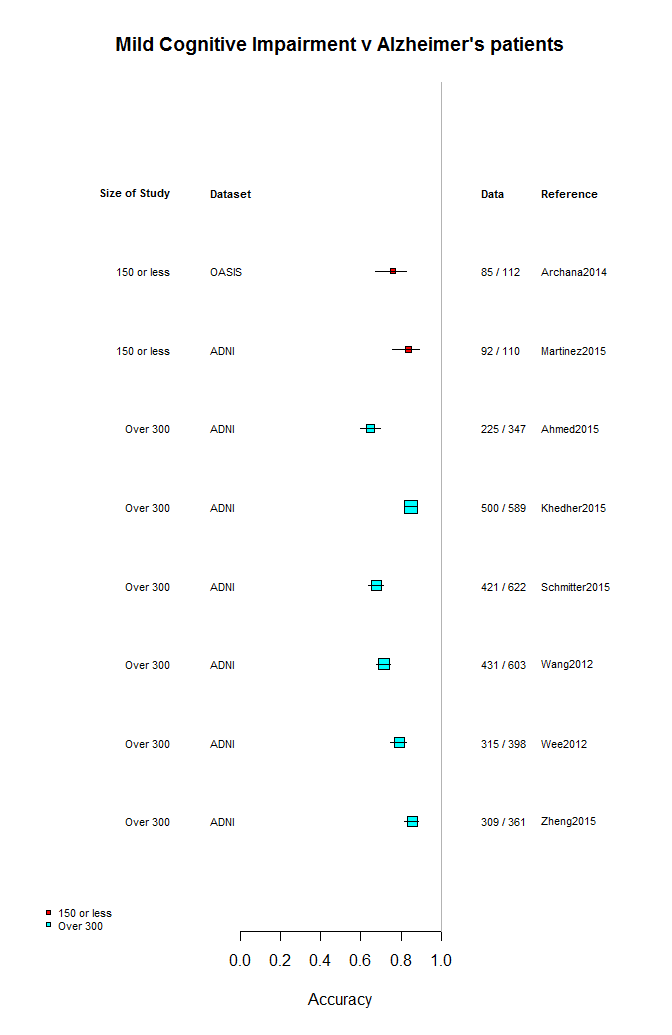
Figure S4 continued – Forest plot of accuracy of studies for differentiating different cognitive states ordered by study size, 2^nd^ page.
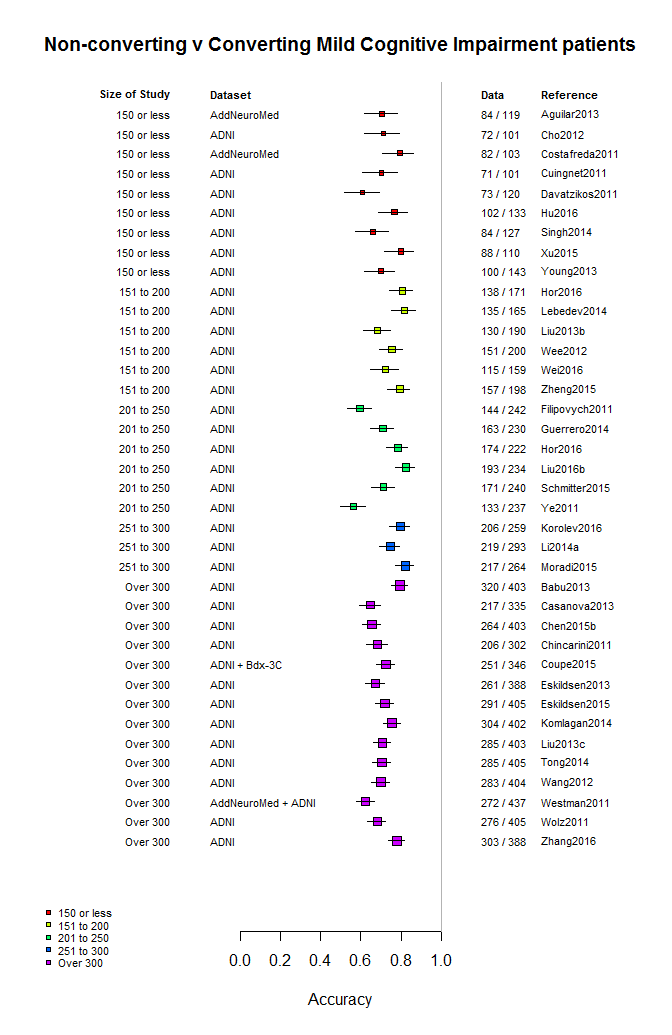


**References**

Aggarwal, N., et al. (2015). "3d discrete wavelet transform for computer aided diagnosis of Alzheimer's disease using t1-weighted brain MRI." International Journal of Imaging Systems and Technology **25**: 179-190.

Aguilar, C., et al. (2013). "Different multivariate techniques for automated classification of MRI data in Alzheimer's disease and mild cognitive impairment." Psychiatry Research: Neuroimaging **212**: 89-98.

Ahmed, O. B., et al. (2015). "Alzheimer's disease diagnosis on structural MR images using circular harmonic functions descriptors on hippocampus and posterior cingulate cortex." Computerized Medical Imaging and Graphics **44**: 13-25.

Anagnostopoulos, C.-N., et al. (2013). Classification models for Alzheimer's disease detection. International Conference on Engineering Applications of Neural Networks**:** 193-202.

Archana, M. and S. Ramakrishnan (2014). Detection of Alzheimer disease in MR images using structure tensor. 2014 36th Annual International Conference of the IEEE Engineering in Medicine and Biology Society**:** 1043-1046.

Babu, G. S., et al. (2013). Meta-cognitive q-Gaussian RBF network for binary classification: Application to mild cognitive impairment (MCI). Neural Networks (IJCNN), The 2013 International Joint Conference on**:** 1-8.

Beheshti, I., et al. (2015). "Probability distribution function-based classification of structural MRI for the detection of Alzheimer's disease." Computers in biology and medicine **64**: 208-216.

Casanova, R., et al. (2013). "Alzheimer's disease risk assessment using large-scale machine learning methods." PLoS ONE **8**: e77949.

Chaddad, A., et al. (2016). Local discriminative characterization of MRI for Alzheimer's disease. 2016 IEEE 13th International Symposium on Biomedical Imaging (ISBI), Institute of Electrical and Electronics Engineers (IEEE).

Chen, L., et al. (2015). Identification of Cerebral Small Vessel Disease Using Multiple Instance Learning. International Conference on Medical Image Computing and Computer-Assisted Intervention**:** 523-530.

Chen, X., et al. (2015). Group Sparse Representation for Prediction of MCI Conversion to AD. International Conference on Intelligent Computing**:** 510-519.

Chen, Y. and T. D. Pham (2013). "Development of a brain MRI-based hidden Markov model for dementia recognition." Biomedical engineering online **12**: 1.

Chincarini, A., et al. (2011). "Local MRI analysis approach in the diagnosis of early and prodromal Alzheimer's disease." Neuroimage **58**: 469-480.

Cho, Y., et al. (2012). "Individual subject classification for Alzheimer's disease based on incremental learning using a spatial frequency representation of cortical thickness data." Neuroimage **59**: 2217-2230.

Costafreda, S. G., et al. (2011). "Automated hippocampal shape analysis predicts the onset of dementia in mild cognitive impairment." NeuroImage **56**: 212-219.

Coupé, P., et al. (2015). "Detection of Alzheimer's disease signature in MR images seven years before conversion to dementia: Toward an early individual prognosis." Human Brain Mapping **36**: 4758-4770.

Cui, Y., et al. (2012). "Automated detection of amnestic mild cognitive impairment in community-dwelling elderly adults: a combined spatial atrophy and white matter alteration approach." Neuroimage **59**: 1209-1217.

Cuingnet, R., et al. (2011). "Automatic classification of patients with Alzheimer's disease from structural MRI: a comparison of ten methods using the ADNI database." neuroimage **56**: 766-781.

Cuingnet, R., et al. (2013). "Spatial and Anatomical Regularization of SVM: A General Framework for Neuroimaging Data." IEEE Transactions on Pattern Analysis and Machine Intelligence **35**: 682-696.

Davatzikos, C., et al. (2011). "Prediction of MCI to AD conversion, via MRI, CSF biomarkers, and pattern classification." Neurobiology of aging **32**: 2322--e2319.

Ding, Y., et al. (2015). Classification of Alzheimer's disease based on the combination of morphometric feature and texture feature. Bioinformatics and Biomedicine (BIBM), 2015 IEEE International Conference on**:** 409-412.

Dou, Q., et al. (2016). "Automatic Detection of Cerebral Microbleeds From MR Images via 3D Convolutional Neural Networks." IEEE Trans Med Imaging **35**: 1182-1195.

Dubey, R., et al. (2014). "Analysis of sampling techniques for imbalanced data: An n= 648 ADNI study." NeuroImage **87**: 220-241.

Dyrba, M., et al. (2012). Combining DTI and MRI for the automated detection of Alzheimer's disease using a large European multicenter dataset. International Workshop on Multimodal Brain Image Analysis**:** 18-28.

Erus, G., et al. (2014). "Individualized statistical learning from medical image databases: Application to identification of brain lesions." Medical image analysis **18**: 542-554.

Eskildsen, S. F., et al. (2015). "Structural imaging biomarkers of Alzheimer's disease: predicting disease progression." Neurobiology of aging **36**: S23--S31.

Eskildsen, S. F., et al. (2013). "Prediction of Alzheimer's disease in subjects with mild cognitive impairment from the ADNI cohort using patterns of cortical thinning." Neuroimage **65**: 511-521.

Fazlollahi, A., et al. (2015). "Computer-aided detection of cerebral microbleeds in susceptibility-weighted imaging." Computerized Medical Imaging and Graphics **46**: 269-276.

Filipovych, R., et al. (2011). "Semi-supervised pattern classification of medical images: application to mild cognitive impairment (MCI)." NeuroImage **55**: 1109-1119.

Fiot, J.-B., et al. (2013). "Efficient brain lesion segmentation using multi-modality tissue-based feature selection and support vector machines." International Journal for Numerical Methods in Biomedical Engineering **29**: 905-915.

Ghafaryasl, B., et al. (2012). A computer aided detection system for cerebral microbleeds in brain MRI. 2012 9th IEEE International Symposium on Biomedical Imaging (ISBI)**:** 138-141.

Granziera, C., et al. (2015). "A multi-contrast MRI study of microstructural brain damage in patients with mild cognitive impairment." NeuroImage: Clinical **8**: 631-639.

Gray, K. R., et al. (2013). "Random forest-based similarity measures for multi-modal classification of Alzheimer's disease." NeuroImage **65**: 167-175.

Griffanti, L., et al. (2016). "BIANCA (Brain Intensity AbNormality Classification Algorithm): A new tool for automated segmentation of white matter hyperintensities." NeuroImage **141**: 191-205.

Guerrero, R., et al. (2014). "Manifold population modeling as a neuro-imaging biomarker: Application to ADNI and ADNI-GO." NeuroImage **94**: 275-286.

Guo, D., et al. (2015). "Automated lesion detection on MRI scans using combined unsupervised and supervised methods." BMC medical imaging **15**: 1.

Herrera, L. J., et al. (2013). Classification of MRI Images for Alzheimer's Disease Detection. 2013 International Conference on Social Computing, Institute of Electrical and Electronics Engineers (IEEE).

Hinrichs, C., et al. (2009). "Spatially augmented LPboosting for AD classification with evaluations on the ADNI dataset." NeuroImage **48**: 138-149.

Hinrichs, C., et al. (2011). "Predictive markers for AD in a multi-modality framework: An analysis of MCI progression in the ADNI population." NeuroImage **55**: 574-589.

Hor, S. and M. Moradi (2016). "Learning in data-limited multimodal scenarios: Scandent decision forests and tree-based features." Medical Image Analysis **34**: 30-41.

Hu, K., et al. (2016). "Multi-scale features extraction from baseline structure MRI for MCI patient classification and AD early diagnosis." Neurocomputing **175**: 132-145.

Illan, I. A., et al. (2014). "Spatial component analysis of MRI data for Alzheimer's disease diagnosis: a Bayesian network approach." Front. Comput. Neurosci. **8**.

Ithapu, V., et al. (2014). "Extracting and summarizing white matter hyperintensities using supervised segmentation methods in Alzheimer's disease risk and aging studies." Human Brain Mapping: n/a--n/a.

Jiang, Q. and J. Shi (2014). Sparse kernel entropy component analysis for dimensionality reduction of neuroimaging data. 2014 36th Annual International Conference of the IEEE Engineering in Medicine and Biology Society, Institute of Electrical and Electronics Engineers (IEEE).

Jie, B., et al. (2014). "Manifold regularized multitask feature learning for multimodality disease classification." Human Brain Mapping **36**: 489-507.

Khedher, L., et al. (2015). "Early diagnosis of Alzheimer's disease based on partial least squares, principal component analysis and support vector machine using segmented MRI images." Neurocomputing **151**: 139-150.

Koikkalainen, J., et al. (2016). "Differential diagnosis of neurodegenerative diseases using structural MRI data." NeuroImage: Clinical **11**: 435-449.

Komlagan, M., et al. (2014). Anatomically Constrained Weak Classifier Fusion for Early Detection of Alzheimer's Disease. International Workshop on Machine Learning in Medical Imaging**:** 141-148.

Korolev, I. O., et al. (2016). "Predicting Progression from Mild Cognitive Impairment to Alzheimer's Dementia Using Clinical, MRI, and Plasma Biomarkers via Probabilistic Pattern Classification." PLoS ONE **11**: e0138866.

Krashenyi, I., et al. (2016). "Fuzzy Computer-Aided Alzheimer’s Disease Diagnosis Based on MRI Data." Current Alzheimer Research **13**: 545-556.

Lebedev, A. V., et al. (2014). "Random Forest ensembles for detection and prediction of Alzheimer's disease with a good between-cohort robustness." NeuroImage: Clinical **6**: 115-125.

Li, H., et al. (2014). "Hierarchical Interactions Model for Predicting Mild Cognitive Impairment (MCI) to Alzheimer's Disease (AD) Conversion." PLoS ONE **9**: e82450.

Li, L., et al. (2010). Detection of Mild Cognitive Impairment Using Image Differences and Clinical Features. 2010 IEEE International Conference on BioInformatics and BioEngineering, Institute of Electrical and Electronics Engineers (IEEE).

Li, M., et al. (2014). "An Efficient Approach for Differentiating Alzheimer's Disease from Normal Elderly Based on Multicenter MRI Using Gray-Level Invariant Features." PLoS ONE **9**: e105563.

Li, Y., et al. (2015). Classification of Alzheimer's Disease Based on Multiple Anatomical Structures' Asymmetric Magnetic Resonance Imaging Feature Selection. Neural Information Processing, Springer Science Business Media**:** 280-289.

Liu, F., et al. (2013). High-order graph matching based feature selection for Alzheimer's disease identification. International Conference on Medical Image Computing and Computer-Assisted Intervention**:** 311-318.

Liu, F., et al. (2014). "Multiple Kernel Learning in the Primal for Multimodal Alzheimer's Disease Classification." IEEE Journal of Biomedical and Health Informatics **18**: 984-990.

Liu, M., et al. (2016). "Inherent Structure-Based Multiview Learning With Multitemplate Feature Representation for Alzheimer's Disease Diagnosis." IEEE Transactions on Biomedical Engineering **63**: 1473-1482.

Liu, M., et al. (2013). "Identifying Informative Imaging Biomarkers via Tree Structured Sparse Learning for AD Diagnosis." Neuroinform **12**: 381-394.

Liu, S., et al. (2016). "Cross-View Neuroimage Pattern Analysis in Alzheimer's Disease Staging." Frontiers in Aging Neuroscience **8**.

Liu, S., et al. (2015). "Multimodal Neuroimaging Feature Learning for Multiclass Diagnosis of Alzheimer's Disease." IEEE Transactions on Biomedical Engineering **62**: 1132-1140.

Liu, X., et al. (2013). "Locally linear embedding (LLE) for MRI based Alzheimer's disease classification." NeuroImage **83**: 148-157.

Luchtenberg, A., et al. (2014). Early detection of Alzheimer's disease using histograms in a dissimilarity-based classification framework. SPIE Medical Imaging**:** 903502.

Maier, O., et al. (2015). "Classifiers for Ischemic Stroke Lesion Segmentation: A Comparison Study." PLoS ONE **10**: e0145118.

Martinez-Murcia, F., et al. (2016). "A Spherical Brain Mapping of MR Images for the Detection of Alzheimer's Disease." CAR **13**: 575-588.

Martinez-Torteya, A., et al. (2015). "Improved Diagnostic Multimodal Biomarkers for Alzheimer's Disease and Mild Cognitive Impairment." BioMed Research International **2015**: 1-11.

McEvoy, L. K., et al. (2009). "Alzheimer Disease: Quantitative Structural Neuroimaging for Detection and Prediction of Clinical and Structural Changes in Mild Cognitive Impairment." Radiology **251**: 195-205.

Moradi, E., et al. (2015). "Machine learning framework for early MRI-based Alzheimer's conversion prediction in MCI subjects." NeuroImage **104**: 398-412.

Morgado, P. M. and M. Silveira (2015). "Minimal neighborhood redundancy maximal relevance: Application to the diagnosis of Alzheimer's disease." Neurocomputing **155**: 295-308.

Nho, K., et al. (2010). Automatic prediction of conversion from mild cognitive impairment to probable Alzheimer's disease using structural magnetic resonance imaging. AMIA Annual Symposium Proceedings. **2010:** 542.

Oppedal, K., et al. (2015). "Classifying Dementia Using Local Binary Patterns from Different Regions in Magnetic Resonance Images." International Journal of Biomedical Imaging **2015**: 1-14.

Plocharski, M. and L. R. Østergaard (2016). "Extraction of sulcal medial surface and classification of Alzheimer's disease using sulcal features." Computer Methods and Programs in Biomedicine **133**: 35-44.

Rao, A., et al. (2011). Classification of Alzheimer's Disease from structural MRI using sparse logistic regression with optional spatial regularization. 2011 Annual International Conference of the IEEE Engineering in Medicine and Biology Society, Institute of Electrical and Electronics Engineers (IEEE).

Rueda, A., et al. (2014). "Extracting Salient Brain Patterns for Imaging-Based Classification of Neurodegenerative Diseases." IEEE Trans Med Imaging **33**: 1262-1274.

Savio, A. and M. GrañA (2013). "Deformation based feature selection for computer aided diagnosis of Alzheimer's disease." Expert Systems with Applications **40**: 1619-1628.

Schmitter, D., et al. (2015). "An evaluation of volume-based morphometry for prediction of mild cognitive impairment and Alzheimer's disease." NeuroImage: Clinical **7**: 7-17.

Schouten, T. M., et al. (2016). "Combining anatomical, diffusion, and resting state functional magnetic resonance imaging for individual classification of mild and moderate Alzheimer's disease." NeuroImage: Clinical **11**: 46-51.

Shi, Y., et al. (2014). Joint Coupled-Feature Representation and Coupled Boosting for AD Diagnosis. 2014 IEEE Conference on Computer Vision and Pattern Recognition, Institute of Electrical and Electronics Engineers (IEEE).

Singh, N., et al. (2014). "Quantifying anatomical shape variations in neurological disorders." Medical Image Analysis **18**: 616-633.

Spulber, G., et al. (2013). "An MRI-based index to measure the severity of Alzheimer's disease-like structural pattern in subjects with mild cognitive impairment." J Intern Med **273**: 396-409.

Suk, H.-I., et al. (2015). "Deep sparse multi-task learning for feature selection in Alzheimer's disease diagnosis." Brain Structure and Function **221**: 2569-2587.

Tong, T., et al. (2014). "Multiple instance learning for classification of dementia in brain MRI." Medical Image Analysis **18**: 808-818.

Uchiyama, Y., et al. (2014). "Eigenspace Template Matching for Detection of Lacunar Infarcts on MR Images." Journal of Digital Imaging **28**: 116-122.

Varol, E., et al. (2012). Feature ranking based nested support vector machine ensemble for medical image classification. 2012 9th IEEE International Symposium on Biomedical Imaging (ISBI), Institute of Electrical and Electronics Engineers (IEEE).

Vemuri, P., et al. (2008). "Alzheimer's disease diagnosis in individual subjects using structural MR images: Validation studies." NeuroImage **39**: 1186-1197.

Vemuri, P., et al. (2011). "Antemortem differential diagnosis of dementia pathology using structural MRI: Differential-STAND." NeuroImage **55**: 522-531.

Vos, P. C., et al. (2013). Automatic detection and segmentation of ischemic lesions in computed tomography images of stroke patients. Medical Imaging 2013: Computer-Aided Diagnosis. C. L. Novak and S. Aylward, SPIE-Intl Soc Optical Eng.

Wachinger, C. and M. Reuter (2016). "Domain adaptation for Alzheimer's disease diagnostics." NeuroImage **139**: 470-479.

Wang, B., et al. (2015). A hierarchical model for identifying mild cognitive impairment. 2015 11th International Conference on Natural Computation (ICNC), Institute of Electrical and Electronics Engineers (IEEE).

Wang, J., et al. (2016). "A Comparison of Magnetic Resonance Imaging and Neuropsychological Examination in the Diagnostic Distinction of Alzheimer's Disease and Behavioral Variant Frontotemporal Dementia." Frontiers in Aging Neuroscience **8**.

Wang, Y., et al. (2012). Groupwise segmentation improves neuroimaging classification accuracy. International Workshop on Multimodal Brain Image Analysis**:** 185-193.

Wee, C.-Y., et al. (2012). "Prediction of Alzheimer's disease and mild cognitive impairment using cortical morphological patterns." Human Brain Mapping **34**: 3411-3425.

Wei, R., et al. (2016). "Prediction of Conversion from Mild Cognitive Impairment to Alzheimer's Disease Using MRI and Structural Network Features." Frontiers in Aging Neuroscience **8**.

Westman, E., et al. (2011). "AddNeuroMed and ADNI: Similar patterns of Alzheimer's atrophy and automated MRI classification accuracy in Europe and North America." NeuroImage **58**: 818-828.

Wolz, R., et al. (2011). "Multi-Method Analysis of MRI Images in Early Diagnostics of Alzheimer's Disease." PLoS ONE **6**: e25446.

Xie, Y., et al. (2015). "Identification of Amnestic Mild Cognitive Impairment Using Multi-Modal Brain Features: A Combined Structural MRI and Diffusion Tensor Imaging Study." Journal of Alzheimer?s Disease **47**: 509-522.

Xu, L., et al. (2015). "Multi-modality sparse representation-based classification for Alzheimer's disease and mild cognitive impairment." Computer Methods and Programs in Biomedicine **122**: 182-190.

Yang, W., et al. (2014). ICA image feature extraction for improving diagnosis of Alzheimer's disease and mild cognitive impairment. 2014 10th International Conference on Natural Computation (ICNC), Institute of Electrical and Electronics Engineers (IEEE).

Ye, D. H., et al. (2011). Semi-supervised Pattern Classification: Application to Structural MRI of Alzheimer's Disease. 2011 International Workshop on Pattern Recognition in NeuroImaging, Institute of Electrical and Electronics Engineers (IEEE).

Ye, T., et al. (2015). Discriminative Multi-task Feature Selection for Multi-modality Based AD/MCI Classification. 2015 International Workshop on Pattern Recognition in NeuroImaging, Institute of Electrical and Electronics Engineers (IEEE).

Young, J., et al. (2013). "Accurate multimodal probabilistic prediction of conversion to Alzheimer's disease in patients with mild cognitive impairment." NeuroImage: Clinical **2**: 735-745.

Zhang, D., et al. (2011). "Multimodal classification of Alzheimer's disease and mild cognitive impairment." NeuroImage **55**: 856-867.

Zhang, J., et al. (2016). Applying sparse coding to surface multivariate tensor-based morphometry to predict future cognitive decline. 2016 IEEE 13th International Symposium on Biomedical Imaging (ISBI), Institute of Electrical and Electronics Engineers (IEEE).

Zhang, Y. and S. Wang (2015). "Detection of Alzheimer's disease by displacement field and machine learning." PeerJ **3**: e1251.

Zhang, Y., et al. (2015). "Detection of Alzheimer's disease and mild cognitive impairment based on structural volumetric MR images using 3D-DWT and WTA-KSVM trained by PSOTVAC." Biomedical Signal Processing and Control **21**: 58-73.

Zheng, W., et al. (2015). "Novel Cortical Thickness Pattern for Accurate Detection of Alzheimer's Disease." Journal of Alzheimer?s Disease **48**: 995-1008.

Zheng, X., et al. (2016). Multi-modality stacked deep polynomial network based feature learning for Alzheimer's disease diagnosis. 2016 IEEE 13th International Symposium on Biomedical Imaging (ISBI), Institute of Electrical and Electronics Engineers (IEEE).

Zhou, Q., et al. (2014). "An Optimal Decisional Space for the Classification of Alzheimer's Disease and Mild Cognitive Impairment." IEEE Transactions on Biomedical Engineering **61**: 2245-2253.

Zhu, J. and J. Shi (2014). Hessian regularization based semi-supervised dimensionality reduction for neuroimaging data of Alzheimer's disease. 2014 IEEE 11th International Symposium on Biomedical Imaging (ISBI), Institute of Electrical and Electronics Engineers (IEEE).

Zhu, J., et al. (2014). Co-training based semi-supervised classification of Alzheimer's disease. 2014 19th International Conference on Digital Signal Processing, Institute of Electrical and Electronics Engineers (IEEE).

Zhu, X., et al. (2015). "Canonical feature selection for joint regression and multi-class identification in Alzheimer's disease diagnosis." Brain Imaging and Behavior **10**: 818-828.

Zhu, X., et al. (2014). A Novel Multi-relation Regularization Method for Regression and Classification in AD Diagnosis. Medical Image Computing and Computer-Assisted Intervention MICCAI 2014, Springer Science Business Media**:** 401-408.
