# Supplementary material for: Machine learning of neuroimaging for assisted diagnosis of cognitive impairment and dementia: A systematic review
Source: Alzheimers Dement (Amst). 2018 Aug 11;10:519–35. doi: 10.1016/j.dadm.2018.07.004 (PMC6197752; doi:10.1016/j.dadm.2018.07.004)
Supplement: Supplementary Prospero [file mmc2.pdf]

Accuracy of machine learning for the automated detection of brain imaging abnormalities in dementia, stroke and pathological ageing: a systematic review

Enrico Pellegrini, Victor Gonzalez Castro, Grant Mair, Maria Valdes Hernandez, Lucia Ballerini, Francesca Chappell, Devasuda Anblagan, Joanna Wardlaw

Citation

Enrico Pellegrini, Victor Gonzalez Castro, Grant Mair, Maria Valdes Hernandez, Lucia Ballerini, Francesca Chappell, Devasuda Anblagan, Joanna Wardlaw. Accuracy of machine learning for the automated detection of brain imaging abnormalities in dementia, stroke and pathological ageing: a systematic review. PROSPERO 2016 CRD42016037332 Available from: http://www.crd.york.ac.uk/PROSPERO/display_record.php?ID=CRD42016037332

Review question

What machine learning approaches have been applied in studies investigating structural brain imaging in stroke, dementia, and pathological ageing populations?
What is the level of accuracy (sensitivity / specificity) that can currently be achieved by machine learning techniques in the automatic diagnosis of dementia?
What level of accuracy can be achieved in the automatic detection / segmentation of brain lesions associated with stroke, dementia and pathological ageing?
What types of features have been studied the most? Which features seem the most promising?
Have these techniques been tested in a clinical environment? Can they be generalized in the case of sets of images that were acquired with different scanners (different resolution/ scan parameters/ sequences, etc)?

Searches

Databases:
PubMed/MEDLINE
Scopus
IEEE Xplore Digital Library
Science Direct
ACM Digital Library
Web of Science
Restrictions:
Year of publication >= 2006.

Types of study to be included

There are no restrictions on the types of study design eligible for inclusion as far as the criteria on dataset size and quality are met.

Condition or domain being studied

The diseases being studied are dementia (Alzheimer's, vascular, frontotemporal, Lewy bodies, etc.), stroke and brain lesions associated with pathological ageing (white matter lesions, cerebral microbleeds, enlarged perivascular spaces, etc.).

Participants/population

Inclusion: adults with dementia, stroke, or brain lesions associated with pathological ageing (as diagnosed using any recognised diagnostic criteria). Structural MRI or CT scans have to be available for each participant.

Intervention(s), exposure(s)

Not applicable.

Comparator(s)/control
Not applicable.

Context

Primary outcome(s)
Accuracy (sensitivity / specificity) of machine learning techniques for automated disease diagnosis, lesion detection or segmentation.

Secondary outcome(s)
Additional metrics on technique accuracy and / or correlation with clinical outcome, if available.

Data extraction (selection and coding)

Risk of bias (quality) assessment
Included studies will be graded by researchers according to the QUADAS-2checklist (http://www.bristol.ac.uk/media-library/sites/quadas/migrated/documents/quadas2.pdf). The results will be reported in the review and in the discussion on the data synthesis.

Strategy for data synthesis
We will provide a narrative synthesis of the findings and the applicability of the included studies. Additionally, the studies will be divided in subgroups according to their main target (e.g., automatic diagnosis of dementia, automatic segmentation of stroke lesions, etc.). A quantitative meta-analysis (including forest plots) for summary estimates of sensitivity and specificity with 95% CIs of the different subgroups is planned to be carried out.

Analysis of subgroups or subsets
Included studies will be analysed separately according to the their main target. Specifically, the subgroups are envisioned to be:
1. Studies on automatic classification of dementia vs healthy control. Additional subgroups, might involve classification of mild cognitive impairment progression and multi-class classification.
2. Studies on automatic detection and/or segmentation of stroke and related lesions.
3. Studies on detection and/or segmentation of brain lesions associated with pathological ageing.

Contact details for further information
Dr Pellegrini
e.pellegrini@ed.ac.uk

Organisational affiliation of the review
University of Edinburgh
http://www.ed.ac.uk/clinical-brain-sciences

Review team members and their organisational affiliations
Dr Enrico Pellegrini.
Dr Victor Gonzalez Castro.
Dr Grant Mair.
Dr Maria Valdes Hernandez.
Dr Lucia Ballerini.
Dr Francesca Chappell.
Dr Devasuda Anblagan.
Professor Joanna Wardlaw.

Collaborators
Dr Susana Munoz-Maniega.
Dr Amos Storkley.

Anticipated or actual start date
18 April 2016

Anticipated completion date
18 October 2016

Funding sources/sponsors
EPSRC grant EP/M005976/1.
Row Fogo Charitable Trust (Grants Nos. R35865 and R43412).
MRC grant G0701120.

Conflicts of interest
None known

Language
English

Country
Scotland

Stage of review
Review_Ongoing

Subject index terms status
Subject indexing assigned by CRD

Subject index terms
Aging; Dementia; Humans; Machine Learning; Neuroimaging; Stroke

Date of registration in PROSPERO
16 May 2016

Date of publication of this version
06 June 2016

Revision note for this version
The QUADAS-2 checklist was chosen instead of the STARD one after further discussion among the research team as deemed more relevant.

Details of any existing review of the same topic by the same authors

Stage of review at time of this submission

Stage	Started	Completed
Preliminary searches	No	Yes
Piloting of the study selection process	No	No
Formal screening of search results against eligibility criteria	No	No
Data extraction	No	No
Risk of bias (quality) assessment	No	No
Data analysis	No	No

Revision note

The QUADAS-2 checklist was chosen instead of the STARD one after further discussion among the research team as deemed more relevant.

Versions

16 May 2016
06 June 2016

PROSPERO
This information has been provided by the named contact for this review. CRD has accepted this information in good faith and registered the review in PROSPERO. CRD bears no responsibility or liability for the content of this registration record, any associated files or external websites.
